# Simultaneous Event-Triggered Fault Detection and Estimation for Stochastic Systems Subject to Deception Attacks

**DOI:** 10.3390/s18020321

**Published:** 2018-01-23

**Authors:** Yunji Li, QingE Wu, Li Peng

**Affiliations:** 1 Key Laboratory of Advanced Process Control for Light Industry (Ministry of Education), Jiangnan University, Wuxi 214122, China; 7141905009@vip.jiangnan.edu.cn (Y.L.); pengli@jiangnan.edu.cn (L.P.); 2 School of Electrical and Information Engineering, Zhengzhou University of Light Industry, Zhengzhou 450002, China

**Keywords:** fault detection and estimation, event-triggered transmission scheme, coordinate transformation

## Abstract

In this paper, a synthesized design of fault-detection filter and fault estimator is considered for a class of discrete-time stochastic systems in the framework of event-triggered transmission scheme subject to unknown disturbances and deception attacks. A random variable obeying the Bernoulli distribution is employed to characterize the phenomena of the randomly occurring deception attacks. To achieve a fault-detection residual is only sensitive to faults while robust to disturbances, a coordinate transformation approach is exploited. This approach can transform the considered system into two subsystems and the unknown disturbances are removed from one of the subsystems. The gain of fault-detection filter is derived by minimizing an upper bound of filter error covariance. Meanwhile, system faults can be reconstructed by the remote fault estimator. An recursive approach is developed to obtain fault estimator gains as well as guarantee the fault estimator performance. Furthermore, the corresponding event-triggered sensor data transmission scheme is also presented for improving working-life of the wireless sensor node when measurement information are aperiodically transmitted. Finally, a scaled version of an industrial system consisting of local PC, remote estimator and wireless sensor node is used to experimentally evaluate the proposed theoretical results. In particular, a novel fault-alarming strategy is proposed so that the real-time capacity of fault-detection is guaranteed when the event condition is triggered.

## 1. Introduction

Wireless sensor networks (WSNs) have grown rapidly in the past decades and found wide applications in the areas of industrial process, smart building, health care and battlefield surveillance, etc. [1,2]. In those applications, data-transmission usually communicates over a wireless channel. Replacing old batteries without energy of wireless sensors is always a costly operation or even impossible [3]. In addition, the capacity of a wireless channel normally varies with external environment. This time-varying property can impact the overall dynamic system performance. Consequently, less data-transmission between the sensor and the remote state estimator (or actuator) can significantly prolong the lifetime of the sensors. Event-triggered transmission schemes provide an inspiring opportunity to a trade-off between energy efficiency and system performance.

In parallel with the quiet evolution of WSNs technologies, the network security problem has recently become an emerging topic of research from the defenders’ perspectives. Due to the unprotected and shared property of a wireless communication link, the exchanged information can be easily exploited by adversaries. It is worth mentioning that a deception attack is one of the most dangerous attack behaviors which can maliciously degrade network reliability through arbitrarily injecting the false data information. Using the techniques of variance-constrained and Lyapunov stability theories, some excellent results concerning security control and estimation problems have been reported in [4,5,6]. For instant, a security-guaranteed filter was designed in [4] for nonlinear stochastic time-delay systems with randomly occurring sensor saturations and deception attacks, where a new concept of mean-square security domain was introduced to quantify the security degree. In [5], a variance-constrained distributed filtering was presented for time-varying systems subject to multiplicative noises and deception attacks over sensor networks. Reference [6] addressed a problem of observer-based event-triggered consensus control for a class of discrete-time multi-agent systems with lossy sensors and cyber-attacks, where a dynamic output feedback controller was derived such that the prescribed security in probability was guaranteed while obtaining an upper bound of the quadratic cost criterion.

For the purpose of increasing the safety and reliability in modern dynamical system, model-based fault diagnosis has been promising research and application topics. In the model-based fault diagnosis, the observer-based strategy is often viewed as one of the most effective methods for fault diagnosis. So far, the issues of observer-based fault diagnosis for dynamic system have received a great deal of attentions from many researchers, and lots of outstanding results were made: [7,8,9,10,11,12,13,14,15,16,17]. Similar to event-triggered control and estimation problems [18,19,20], the event-triggered data transmission scheme could also be applied to fault diagnosis purposes. An event-triggered fault-detection filter was established in [21] for discrete-time systems with nonlinear perturbation subject to transmission delay, where the designed filter parameters were given through solving some linear matrix inequalities. Literature [22] studied fault detection and isolation using event-triggered sensor data transmission strategy. Its feature was that the proposed fault detection filter was presented based on $l_{1}$, $H_{-}$ and $H_{\infty }$, and further applied to underwater robotic platforms. A polynomial fuzzy fault-detection filter was designed in [23] for nonlinear discrete-time systems under an event-triggered data transmission framework, where the asymptotically stable of the polynomial fault-detection residual system was guaranteed while satisfying the desired performance criteria.

The aforementioned studies have proposed some results to show the effectiveness of event-triggered scheme in fault diagnosis. However, an event-triggered fault detection and estimation problem have not been fully investigated yet especially when the system is subject to deception attacks in a possibly random way. On the other hand, the sensibility and real-time capacity of fault detection were neglected in the existing literature. Due to the considered event-triggered scheme adopting an aperiodic fashion to data-transmission, remote fault-detection filter can not detect in real time when a fault occurs. Furthermore, a fault-detection residual may further become insensitive, because unknown disturbances and faults are often coupled in practical systems, it is difficult to distinguish among them. Therefore, the purpose of the paper is to solve the problems we discussed. The main contributions of this paper are summarized as follows:

(1) A synthesized design of fault-detection filter and fault estimator with an event-triggered sensor data transmission scheme in discrete-time stochastic systems is proposed, for the first time, to deal with the phenomena of both unknown disturbances and randomly occurring deception attacks, which reflects the reality closely. The design of the fault-detection filter and the fault estimator is simple due to the utilization of reduce-order subsystems.

(2) Using the coordinate transformation approach, the desired fault-detection residual is sensitive only to faults while insensitive to disturbances. An upper bound of the fault-detection filtering error covariance is minimized at each iteration with proper filtering parameters computed via a recursive algorithm. In particular, the proposed fault-alarming strategy cannot be influenced by the impact of event-triggered data transmission to ensure a real-time capacity of fault detection.

(3) System faults can be robustly estimated using the designed event-triggered remote fault estimator at the same time. Two upper bounds of the state and fault estimation error covariance matrices are obtained in form of Riccati-like difference equations by utilizing the mathematical induction approach. The explicit forms of the fault estimator gains are obtained to minimize such upper bounds through a recursive algorithm. Furthermore, the corresponding event-triggered scheme is given to trade the computation for communication.

(4) A simulation testbed where the terminal voltages of a three-cell battery string are estimated is implemented. Estimation accuracy, fault-alarming strategy and battery life are given for verifying the effectiveness of the proposed theoretical algorithms.

*Nomenclature*: $\mathrm{Prob}\left\{x\right\}$ means the occurrence probability of the event *x*. E(·), Var(·) and tr(·) denote the mathematical expectation, variance and the trace of a matrix, respectively. N and R denote the sets of natural and real numbers, respectively. $R^{m\times n}$ denotes the sets of *m* by *n* real-valued matrices, whereas $R^{n}$ is short for $R^{n\times 1}$. $R_{+}^{n\times n}$ and $R_{++}^{n\times n}$ are the sets of n×n positive semi-definite and positive definite matrices, respectively. When $X\in R_{+}^{n\times n}$, it is simply denoted as X≥0 or X>0 if $X\in R_{++}^{n\times n}$. For $X\in R^{m\times n}$, $X^{T}$ denotes the transpose of *X* and $∥X∥$ denotes the Euclidean norm of *X*. “*I*” denotes a identity matrix with appropriate dimensions. Furthermore, the terms state observer and state estimator are used synonymously in this paper.

## 2. Problem Statement

### 2.1. System Model

Consider the following stochastic linear system defined on $k\in \left[0,\;N-1\right]$:

(1) $$\left\{\begin{array}{c} x_{k+1}=Ax_{k}+Ww_{k}+\tilde{F}f_{k}+Dd_{k}\\ {\bar{y}}_{k}=Cx_{k}+Vv_{k}+\bar{F}f_{k} \end{array}\right.$$

In above equations, the constant subscript “*k*” is a discrete-time index, system state $x_{k}$ is a *n*-dimensional vector. The variables ${\bar{y}}_{k}\in R^{p}$ is the sensor’s measurement signals. The additional terms, fault signals $f_{k}$ is a *q*-dimensional vector to be detected as well as estimated, and $d_{k}$ is bounded external disturbances with dimension of s. The process noises $\left\{w_{k}\right\}$ and the measurement noises $\left\{v_{k}\right\}$ are assumed white and zero-mean sequences with known variance: $E\left(w_{k}w_{k}^{T}\right)=Q_{w}\geq 0$, $E\left(v_{k}v_{k}^{T}\right)=R_{v}>0$ and $E\left(x_{0}x_{0}^{T}\right)=P_{0}>0$, respectively. The adopted mathematical model of randomly occurring deception attacks is described by

(2) $$\left\{\begin{array}{c} {\bar{y}}_{a,k}=-{\bar{y}}_{k}+\beta_{k}\\ y_{k}={\bar{y}}_{k}+\alpha_{k}{\bar{y}}_{a,k} \end{array}\right.$$

where the variables ${\bar{y}}_{a,k}\in R^{p}$ and $y_{k}\in R^{p}$ are the attack signals sent by adversaries and the received measurement signals by the remote estimator, respectively. The non-zero $\beta_{k}$ satisfying $∥\beta_{k}∥\leq \beta$ is an arbitrary limited magnitude signal where the bound β is a known positive scalar that can be estimated through statistical tests or specified by security requirements. The random variable $\alpha_{k}$ is a Bernoulli distributed white sequence with the following probabilities

(3) $$\mathrm{Prob}\left\{\alpha_{k}=1\right\}=\alpha \;\mathrm{and}\;\mathrm{Prob}\left\{\alpha_{k}=0\right\}=\alpha_{1}=1-\alpha$$

As can be seen from (2), it is clear that if a deception attack occurs, i.e., $\alpha_{k}=1$, the remote estimator can only obtain the signals $\beta_{k}$ sent by attackers. If $\alpha_{k}=0$, the actual measurement signals ${\bar{y}}_{k}$ are sent to the remote estimator. In other words, the actual measurement signals received by remote estimator can be rewritten as follows

(4) $$y_{k}=\left(1-\alpha_{k}\right){\bar{y}}_{k}+\alpha_{k}\beta_{k}$$

Throughout this paper, the noise signals $\left\{w_{k}\right\}$, $\left\{v_{k}\right\}$ and the stochastic variable $\alpha_{k}$ are assumed mutually independent. It is further supposed that the matrices *A*, *C*, $\tilde{F}$, $\bar{F}$, *W*, *D* and *V* with appropriate dimensions are known. Furthermore, an event-triggered transmission decision variable $\gamma_{k}$ determines whether the current measurement information is sent to the remote estimator or not. When $\gamma_{k}=1$ indicates that $y_{k}$ is sent out and $\gamma_{k}=0$, otherwise. Therefore, only when $\gamma_{k}=1$, the remote estimator knows the exact value $y_{k}$. Clearly, the corresponding event only happens at each time instant.

**Remark** **1.** In practice, attack detectors are usually viewed as a soft barrier, and there exists some network constraints that should be faced by the adversaries. Such constraints include network load, network congestion, and network transmission rate that are typically randomly fluctuated [5]. This kind of random characteristic from network constraints should be taken into consideration if a comprehensive yet realistic deception attack model is to be established. In addition, such a random nature brings a new challenge when designing our fault-detection filter and fault estimator.

For the above system, two assumptions are first introduced as follows.

**Assumption** **1.** [24]

(5) $$rank\left(C\times D\right)=rank\left(D\right)$$

**Assumption** **2.** [11]

For every complex number ζ with nonnegative real part,

(6) $$rank\left(\left[\begin{array}{ccc} A-\zeta I & \tilde{F} & D\\ C & 0 & 0 \end{array}\right]\right)=n+rank\left(\tilde{F}\right)+rank\left(D\right)$$

### 2.2. Transforming of the System into Two Subsystems

Inspired by [11,24,25], we will adopt the coordinate transformation approach to transform the system into two subsystems: the first subsystem will free from disturbances, but subject to system faults. For the first subsystem, a fault-detection residual based on the derived fault-detection filter will only sensitive to faults; inversely, the desired fault estimator of the second subsystem will include disturbances and system faults, which will robust to disturbances. The transformation results are briefly presented below.

According to [11], Assumption 1 is equivalent to the existence of two non-singular matrices *T* and *S* such that

(7) $$x=T^{-1}\left[\begin{array}{c} {\tilde{x}}^{1}\\ {\tilde{x}}^{2} \end{array}\right],\;\mathrm{and}\;y=S^{-1}\left[\begin{array}{c} {\tilde{y}}^{1}\\ {\tilde{y}}^{2} \end{array}\right]$$

respectively, so that the system can be accordingly transformed into

(8) $$\begin{array}{cc} {\tilde{x}}_{k+1}^{1} & ={\tilde{A}}_{11}{\tilde{x}}_{k}^{1}+{\tilde{A}}_{12}{\tilde{x}}_{k}^{2}+{\tilde{W}}_{1}w_{k}+{\tilde{F}}_{1}f_{k}\\ {\tilde{x}}_{k+1}^{2} & ={\tilde{A}}_{22}{\tilde{x}}_{k}^{2}+{\tilde{A}}_{21}{\tilde{x}}_{k}^{1}+{\tilde{W}}_{2}w_{k}+{\tilde{F}}_{2}f_{k}+{\tilde{D}}_{d}d_{k}\\ y_{k}^{1} & =\left(1-\alpha_{k}\right){\tilde{C}}_{11}{\tilde{x}}_{k}^{1}+\left(1-\alpha_{k}\right){\tilde{V}}_{1}v_{k}+\left(1-\alpha_{k}\right){\bar{F}}_{1}f_{k}+\alpha_{k}S_{1}\beta_{k}\\ y_{k}^{2} & =\left(1-\alpha_{k}\right){\tilde{C}}_{22}{\tilde{x}}_{k}^{2}+\left(1-\alpha_{k}\right){\tilde{V}}_{2}v_{k}+\left(1-\alpha_{k}\right){\bar{F}}_{2}f_{k}+\alpha_{k}S_{2}\beta_{k} \end{array}$$

where

(9) $$TAT^{-1}=\left[\begin{array}{cc} {\tilde{A}}_{11} & {\tilde{A}}_{12}\\ {\tilde{A}}_{21} & {\tilde{A}}_{22} \end{array}\right],\;TW=\left[\begin{array}{c} {\tilde{W}}_{1}\\ {\tilde{W}}_{2} \end{array}\right],\;T\tilde{F}=\left[\begin{array}{c} {\tilde{F}}_{1}\\ {\tilde{F}}_{2} \end{array}\right]$$

(10) $$SCT^{-1}=\left[\begin{array}{cc} {\tilde{C}}_{11} & 0\\ 0 & {\tilde{C}}_{22} \end{array}\right],\;SV=\left[\begin{array}{c} {\tilde{V}}_{1}\\ {\tilde{V}}_{2} \end{array}\right],\;S\bar{F}=\left[\begin{array}{c} {\bar{F}}_{1}\\ {\bar{F}}_{2} \end{array}\right],\;S=\left[\begin{array}{c} S_{1}\\ S_{2} \end{array}\right]$$

Matrix ${\tilde{D}}_{d}$ has full rank and ${\tilde{C}}_{22}$ is invertible. It was shown in [11] that the pair $\left({\tilde{C}}_{11},{\tilde{A}}_{11}\right)$ was detectable if and only if Assumption 2 was satisfied. Two subsystems given by (8) can be rewritten separately as

The 1st subsystem:

(11) $$\begin{array}{cc} {\tilde{x}}_{k+1}^{1} & ={\tilde{A}}_{11}{\tilde{x}}_{k}^{1}+{\tilde{A}}_{12}{\tilde{x}}_{k}^{2}+{\tilde{W}}_{1}w_{k}+{\tilde{F}}_{1}f_{k}\\ y_{k}^{1} & =\left(1-\alpha_{k}\right){\tilde{C}}_{11}{\tilde{x}}_{k}^{1}+\left(1-\alpha_{k}\right){\tilde{V}}_{1}v_{k}+\left(1-\alpha_{k}\right){\bar{F}}_{1}f_{k}+\alpha_{k}S_{1}\beta_{k} \end{array}$$

The 2nd subsystem:

(12) $$\begin{array}{cc} {\tilde{x}}_{k+1}^{2} & ={\tilde{A}}_{22}{\tilde{x}}_{k}^{2}+{\tilde{A}}_{21}{\tilde{x}}_{k}^{1}+{\tilde{W}}_{2}w_{k}+{\hat{F}}_{2}f_{d,k}\\ y_{k}^{2} & =\left(1-\alpha_{k}\right){\tilde{C}}_{22}{\tilde{x}}_{k}^{2}+\left(1-\alpha_{k}\right){\tilde{V}}_{2}v_{k}+\left(1-\alpha_{k}\right)F_{2}f_{d,k}+\alpha_{k}S_{2}\beta_{k} \end{array}$$

where ${\hat{F}}_{2}=\left[\begin{array}{cc} {\tilde{F}}_{2} & {\tilde{D}}_{d} \end{array}\right]$, $F_{2}=\left[\begin{array}{cc} {\bar{F}}_{2} & 0 \end{array}\right]$ and $f_{d,k}=\left[\begin{array}{c} f_{k}\\ d_{k} \end{array}\right]$.

**Remark** **2.** From the Formula (11) and (12), it can be easily seen that the 1st subsystem is not included disturbances, as well as the 2nd subsystem contains both. For the 1st subsystem, a fault-detection filter and fault-alarming strategy will be designed under an event-triggered data transmission framework; for the 2nd subsystem, an event-triggered fault estimator will be derived.

Before giving the main results, the following lemma, which will be useful in this paper, needs to be introduced.

**Lemma** **1.** (Lemma 1 [26]) For any two matrices X and Y with appropriate dimensions, the inequality $XY^{T}+YX^{T}\leq \tau XX^{T}+\tau^{-1}YY^{T}$ holds where τ>0 is a constant scalar.

## 3. Event-Triggered Fault-Detection Strategy Based on Reduce-Order Filter

In this section, a fault-detection system will be presented involving a residual generator, a residual evaluator and fault-alarming strategy under an event-triggered data transmission framework.

### 3.1. Residual Generator

For the residual generation, the following reduce-order filter is constructed as follows

(13) $$\left\{\begin{array}{c} {\hat{\tilde{x}}}_{k+1}^{1}={\tilde{A}}_{11}{\hat{\tilde{x}}}_{k}^{1}+{\tilde{A}}_{12}{\hat{\tilde{x}}}_{k}^{2}+K_{k}^{1}\left({\tilde{y}}_{k}^{1}-{\hat{\tilde{y}}}_{k}^{1}\right)\\ {\hat{\tilde{y}}}_{k}^{1}=\alpha_{1}{\tilde{C}}_{11}{\hat{\tilde{x}}}_{k}^{1} \end{array}\right.$$

where ${\hat{\tilde{x}}}_{k}^{i}$ and ${\hat{\tilde{y}}}_{k}^{i}$ indicate estimated state and output estimation values for each i=1 and 2, respectively. The matrix $K_{k}^{1}$ is the filtering gain to be determined. Denote state estimation errors for each i=1 and 2 as

(14) $$e_{k}^{i}={\tilde{x}}_{k}^{i}-{\hat{\tilde{x}}}_{k}^{i}$$

Then, the corresponding error dynamics of the 1st subsystem without system faults are calculated by subtracting the filtering (13) from the 1st subsystem (11)

(15) $$\begin{array}{cc} e_{k+1}^{1} & ={\tilde{x}}_{k+1}^{1}-{\hat{\tilde{x}}}_{k+1}^{1}\\ & =\left({\tilde{A}}_{11}-\alpha_{1}K_{k}^{1}{\tilde{C}}_{11}\right)e_{k}^{1}+{\tilde{A}}_{12}e_{k}^{2}+{\tilde{W}}_{1}w_{k}-\left(1-\alpha_{k}\right)K_{k}^{1}{\tilde{V}}_{1}v_{k}-\alpha_{k}K_{k}^{1}S_{1}\beta_{k}-\left(\alpha_{k}-\alpha \right)K_{k}^{1}{\tilde{C}}_{11}{\tilde{x}}_{k}^{1} \end{array}$$

Further, let us define error covariance $P_{k}^{i}$ for each i=1 and 2 as

(16) $$P_{k}^{i}=E\left[\left({\tilde{x}}_{k}^{i}-{\hat{\tilde{x}}}_{k}^{i}\right){\left({\tilde{x}}_{k}^{i}-{\hat{\tilde{x}}}_{k}^{i}\right)}^{T}\right]$$

The purpose of this section is to design a form (13) of fault-detection filter for the 1st subsystem (11) subject to deception attacks. More specifically, we are interested in looking for the filtering parameter $K_{k}^{1}$ such that the following requirements are met simultaneously:

(1) A form (13) of fault-detection filter such that an upper bound of the filter error convariance $P_{k}^{1}$ is derived, i.e., there exists a sequence of positive-definite matrices ${\bar{P}}_{k}^{1}\left(0\leq k\leq N-1\right)$ that satisfies

(17) $$E\left[\left({\tilde{x}}_{k}^{1}-{\hat{\tilde{x}}}_{k}^{1}\right){\left({\tilde{x}}_{k}^{1}-{\hat{\tilde{x}}}_{k}^{1}\right)}^{T}\right]\leq {\bar{P}}_{k}^{1}$$

(2) The sequence of upper bound ${\bar{P}}_{k}^{1}$ is minimized by the desired filtering gain $K_{k}^{1}$ through a recursive scheme.

Now, an upper bound of the filtering error convariance is presented for our proposed fault-detection filter in the following theorem.

**Theorem** **1.** *For the 1st subsystem (11) subject to deception attacks in the fault-free case and given $\tau_{j}\;\left(j=1,\;2,\;and\;3\right)$, the filtering error covariance satisfies the following form:*

(18) $$P_{k+1}^{1}\leq {\bar{P}}_{k+1}^{1}$$

*where*

(19) $$\begin{array}{cc} {\bar{P}}_{k+1}^{1} & =\left(1+\tau_{1}+\tau_{2}\alpha \right)\left({\tilde{A}}_{11}-\alpha_{1}K_{k}^{1}{\tilde{C}}_{11}\right){\bar{P}}_{k}^{1}{\left({\tilde{A}}_{11}-\alpha_{1}K_{k}^{1}{\tilde{C}}_{11}\right)}^{T}+\left(1+\tau_{1}^{-1}+\tau_{3}\alpha \right){\tilde{A}}_{12}{\bar{P}}_{k}^{2}{\tilde{A}}_{12}^{T}\\ & +\left(\alpha +\tau_{2}^{-1}\alpha +\tau_{3}^{-1}\alpha \right)\beta^{2}K_{k}^{1}S_{1}S_{1}^{T}{\left(K_{k}^{1}\right)}^{T}+{\tilde{W}}_{1}Q_{w}{\left({\tilde{W}}_{1}\right)}^{T}\\ & +\alpha_{2}K_{k}^{1}{\tilde{C}}_{11}\left({\bar{P}}_{k}^{1}+{\hat{\tilde{x}}}_{k}^{1}{\left({\hat{\tilde{x}}}_{k}^{1}\right)}^{T}\right){\tilde{C}}_{11}^{T}{\left(K_{k}^{1}\right)}^{T}+\alpha_{1}K_{k}^{1}{\tilde{D}}_{v}^{1}R_{v}{\left({\tilde{D}}_{v}^{1}\right)}^{T}{\left(K_{k}^{1}\right)}^{T} \end{array}$$

*$\alpha_{1}=1-\alpha$, $\alpha_{2}=E\left[{\left(\alpha_{k}-\alpha \right)}^{2}\right]=\alpha \left(1-\alpha \right)$ and the initial condition ${\bar{P}}_{0}^{1}=P_{0}^{1}$.*

**Proof.**  Based on the error dynamics of the 1st subsystem (15) and the definition of the filtering error covariance (16), the expression for the error covariance matrix of the 1st subsystem can be expanded as

(20) $$\begin{array}{cc} P_{k+1}^{1} & =E\left[\left({\tilde{x}}_{k}^{1}-{\hat{\tilde{x}}}_{k}^{1}\right){\left({\tilde{x}}_{k}^{1}-{\hat{\tilde{x}}}_{k}^{1}\right)}^{T}\right]\\ & =E\left[\left(\left({\tilde{A}}_{11}-\alpha_{1}K_{k}^{1}{\tilde{C}}_{11}\right)e_{k}^{1}+{\tilde{A}}_{12}e_{k}^{2}+{\tilde{W}}_{1}w_{k}-\left(1-\alpha_{k}\right)K_{k}^{1}{\tilde{V}}_{1}v_{k}-\alpha_{k}K_{k}^{1}S_{1}\beta_{k}-\left(\alpha_{k}-\alpha \right){\tilde{C}}_{11}K_{k}^{1}{\tilde{x}}_{k}^{1}\right)\right.\\ & \times \left.{\left(\left({\tilde{A}}_{11}-\alpha_{1}K_{k}^{1}{\tilde{C}}_{11}\right)e_{k}^{1}+{\tilde{A}}_{12}e_{k}^{2}+{\tilde{W}}_{1}w_{k}-\left(1-\alpha_{k}\right)K_{k}^{1}{\tilde{V}}_{1}v_{k}-\alpha_{k}K_{k}^{1}S_{1}\beta_{k}-\left(\alpha_{k}-\alpha \right){\tilde{C}}_{11}K_{k}^{1}{\tilde{x}}_{k}^{1}\right)}^{T}\right]\\ & =\left({\tilde{A}}_{11}-\alpha_{1}K_{k}^{1}{\tilde{C}}_{11}\right)P_{k}^{1}{\left({\tilde{A}}_{11}-\alpha_{1}K_{k}^{1}{\tilde{C}}_{11}\right)}^{T}+{\tilde{A}}_{12}P_{k}^{2}{\tilde{A}}_{12}^{T}+{\tilde{W}}_{1}Q_{w}{\left({\tilde{W}}_{1}\right)}^{T}+\alpha_{1}K_{k}^{1}\tilde{V_{1}}R_{v}{\left({\tilde{V}}_{1}\right)}^{T}{\left(K_{k}^{1}\right)}^{T}\\ & +\alpha K_{k}^{1}S_{1}\beta_{k}\beta_{k}^{T}S_{1}^{T}{\left(K_{k}^{1}\right)}^{T}+\left({\tilde{A}}_{11}-\alpha_{1}K_{k}^{1}{\tilde{C}}_{11}\right)E\left[e_{k}^{1}{\left(e_{k}^{2}\right)}^{T}\right]{\tilde{A}}_{12}^{T}+{\tilde{A}}_{12}E\left[e_{k}^{2}{\left(e_{k}^{1}\right)}^{T}\right]{\left({\tilde{A}}_{11}-\alpha_{1}K_{k}^{1}{\tilde{C}}_{11}\right)}^{T}\\ & -\alpha \left({\tilde{A}}_{11}-\alpha_{1}K_{k}^{1}{\tilde{C}}_{11}\right)E\left[e_{k}^{1}\beta_{k}^{T}\right]S_{1}^{T}{\left(K_{k}^{1}\right)}^{T}-\alpha K_{k}^{1}S_{1}E\left[\beta_{k}{\left(e_{k}^{1}\right)}^{T}\right]{\left({\tilde{A}}_{11}-\alpha_{1}K_{k}^{1}{\tilde{C}}_{11}\right)}^{T}\\ & -\alpha {\tilde{A}}_{12}E\left[e_{k}^{2}\beta_{k}^{T}\right]S_{1}^{T}{\left(K_{k}^{1}\right)}^{T}-\alpha K_{k}^{1}S_{1}E\left[\beta_{k}{\left(e_{k}^{2}\right)}^{T}\right]{\tilde{A}}_{12}^{T}+\alpha_{2}K_{k}^{1}{\tilde{C}}_{11}E\left[{\tilde{x}}_{k}^{1}{\left({\tilde{x}}_{k}^{1}\right)}^{T}\right]{\tilde{C}}_{11}^{T}{\left(K_{k}^{1}\right)}^{T} \end{array}$$

where $\alpha_{1}=1-\alpha$, $\alpha_{2}=E\left[{\left(\alpha_{k}-\alpha \right)}^{2}\right]=\alpha \left(1-\alpha \right)$. Noticing the fact that $E\left[{\tilde{x}}_{k}^{1}{\left({\tilde{x}}_{k}^{1}\right)}^{T}\right]=P_{k}^{1}+{\hat{\tilde{x}}}_{k}^{1}{\left({\hat{\tilde{x}}}_{k}^{1}\right)}^{T}$, we have

(21) $$\begin{array}{cc} P_{k+1}^{1} & =\left({\tilde{A}}_{11}-\alpha_{1}K_{k}^{1}{\tilde{C}}_{11}\right)P_{k}^{1}{\left({\tilde{A}}_{11}-\alpha_{1}K_{k}^{1}{\tilde{C}}_{11}\right)}^{T}+{\tilde{A}}_{12}P_{k}^{2}{\tilde{A}}_{12}^{T}+{\tilde{W}}_{1}Q_{w}{\left({\tilde{W}}_{1}\right)}^{T}+\alpha_{1}K_{k}^{1}{\tilde{V}}_{1}R_{v}{\left({\tilde{V}}_{1}\right)}^{T}{\left(K_{k}^{1}\right)}^{T}\\ & +\alpha K_{k}^{1}S_{1}\beta_{k}\beta_{k}^{T}S_{1}^{T}{\left(K_{k}^{1}\right)}^{T}+\left({\tilde{A}}_{11}-\alpha_{1}K_{k}^{1}{\tilde{C}}_{11}\right)E\left[e_{k}^{1}{\left(e_{k}^{2}\right)}^{T}\right]{\tilde{A}}_{12}^{T}+{\tilde{A}}_{12}E\left[e_{k}^{2}{\left(e_{k}^{1}\right)}^{T}\right]{\left({\tilde{A}}_{11}-\alpha_{1}K_{k}^{1}{\tilde{C}}_{11}\right)}^{T}\\ & -\alpha \left({\tilde{A}}_{11}-\alpha_{1}K_{k}^{1}{\tilde{C}}_{11}\right)E\left[e_{k}^{1}\beta_{k}^{T}\right]S_{1}^{T}{\left(K_{k}^{1}\right)}^{T}-\alpha K_{k}^{1}S_{1}E\left[\beta_{k}{\left(e_{k}^{1}\right)}^{T}\right]{\left({\tilde{A}}_{11}-\alpha_{1}K_{k}^{1}{\tilde{C}}_{11}\right)}^{T}\\ & -\alpha {\tilde{A}}_{12}E\left[e_{k}^{2}\beta_{k}^{T}\right]S_{1}^{T}{\left(K_{k}^{1}\right)}^{T}-\alpha K_{k}^{1}S_{1}E\left[\beta_{k}{\left(e_{k}^{2}\right)}^{T}\right]{\tilde{A}}_{12}^{T}+\alpha_{2}K_{k}^{1}{\tilde{C}}_{11}\left(P_{k}^{1}+{\hat{\tilde{x}}}_{k}^{1}{\left({\hat{\tilde{x}}}_{k}^{1}\right)}^{T}\right){\tilde{C}}_{11}^{T}{\left(K_{k}^{1}\right)}^{T} \end{array}$$

To obtain an upper bound of $P_{k+1}^{1}$, the following parts of this theorem can be proved by mathematical induction. According to the initial condition, we have ${\bar{P}}_{0}^{1}\geq P_{0}^{1}$. Assume that ${\bar{P}}_{k}^{1}\geq P_{k}^{1}$, and then ${\bar{P}}_{k+1}^{1}\geq P_{k+1}^{1}$ need to be proved. With the help of Lemma 1, it follows from (21) that

(22) $$\begin{array}{cc} P_{k+1}^{1} & \leq \left(1+\tau_{1}+\tau_{2}\alpha \right)\left({\tilde{A}}_{11}-\alpha_{1}K_{k}^{1}{\tilde{C}}_{11}\right)P_{k}^{1}{\left({\tilde{A}}_{11}-\alpha_{1}K_{k}^{1}{\tilde{C}}_{11}\right)}^{T}+\left(1+\tau_{1}^{-1}+\tau_{3}\alpha \right){\tilde{A}}_{12}P_{k}^{2}{\tilde{A}}_{12}^{T}\\ & +\left(\alpha +\tau_{2}^{-1}\alpha +\tau_{3}^{-1}\alpha \right)K_{k}^{1}S_{1}\beta_{k}\beta_{k}^{T}S_{1}^{T}{\left(K_{k}^{1}\right)}^{T}+{\tilde{W}}_{1}Q_{w}{\left({\tilde{W}}_{1}\right)}^{T}\\ & +\alpha_{2}K_{k}^{1}{\tilde{C}}_{11}\left(P_{k}^{1}+{\hat{\tilde{x}}}_{k}^{1}{\left({\hat{\tilde{x}}}_{k}^{1}\right)}^{T}\right){\tilde{C}}_{11}^{T}{\left(K_{k}^{1}\right)}^{T}+\alpha_{1}K_{k}^{1}{\tilde{V}}_{1}R_{v}{\left({\tilde{V}}_{1}\right)}^{T}{\left(K_{k}^{1}\right)}^{T} \end{array}$$

Combining inequality $∥\beta_{k}∥\leq \beta$ into Formula (22) leads to

(23) $$\begin{array}{cc} P_{k+1}^{1} & \leq \left(1+\tau_{1}+\tau_{2}\alpha \right)\left({\tilde{A}}_{11}-\alpha_{1}K_{k}^{1}{\tilde{C}}_{11}\right){\bar{P}}_{k}^{1}{\left({\tilde{A}}_{11}-\alpha_{1}K_{k}^{1}{\tilde{C}}_{11}\right)}^{T}+\left(1+\tau_{1}^{-1}+\tau_{3}\alpha \right){\tilde{A}}_{12}{\bar{P}}_{k}^{2}{\tilde{A}}_{12}^{T}\\ & +\left(\alpha +\tau_{2}^{-1}\alpha +\tau_{3}^{-1}\alpha \right)\beta^{2}K_{k}^{1}S_{1}S_{1}^{T}{\left(K_{k}^{1}\right)}^{T}+{\tilde{W}}_{1}Q_{w}{\left({\tilde{W}}_{1}\right)}^{T}\\ & +\alpha_{2}K_{k}^{1}{\tilde{C}}_{11}\left({\bar{P}}_{k}^{1}+{\hat{\tilde{x}}}_{k}^{1}{\left({\hat{\tilde{x}}}_{k}^{1}\right)}^{T}\right){\tilde{C}}_{11}^{T}{\left(K_{k}^{1}\right)}^{T}+\alpha_{1}K_{k}^{1}{\tilde{V}}_{1}R_{v}{\left({\tilde{V}}_{1}\right)}^{T}{\left(K_{k}^{1}\right)}^{T}={\bar{P}}_{k+1}^{1} \end{array}$$

which implies that inequality (18) is true. ☐

So far, the upper bound of the filtering error covariance has been presented in the above results. We are now in a position to derive the desired filtering gain $K_{k}^{1}$ through minimizing this upper bound at every time instant.

**Theorem** **2.** *Consider the 1st subsystem (11) with deception attacks in the fault-free case. For given $\tau_{j}\;\left(j=1,\;2,\;and\;3\right)$, the filtering gain is given by*

(24) $$\begin{array}{cc} K_{k}^{1} & =\alpha_{1}\left(1+\tau_{1}+\tau_{2}\alpha^{2}\right){\tilde{A}}_{11}{\bar{P}}_{k}^{1}{\tilde{C}}_{11}^{T}\\ & \times {\left(\alpha_{1}^{2}\left(1+\tau_{1}+\tau_{2}\alpha^{2}\right){\tilde{C}}_{11}{\bar{P}}_{k}^{1}{\tilde{C}}_{11}^{T}+\left(\alpha +\tau_{2}^{-1}+\tau_{3}^{-1}\right)\beta^{2}S_{1}S_{1}^{T}+\alpha_{1}{\tilde{D}}_{v}^{1}R_{v}{\left({\tilde{D}}_{v}^{1}\right)}^{T}+\tilde{X}\right)}^{-1} \end{array}$$

*where $\tilde{X}=\alpha_{2}{\tilde{C}}_{11}\left({\bar{P}}_{k}^{1}+{\hat{\tilde{x}}}_{k}^{1}{\left({\hat{\tilde{x}}}_{k}^{1}\right)}^{T}\right){\tilde{C}}_{11}^{T}$. The obtained upper bound ${\bar{P}}_{k+1}^{1}$ of the filtering error covariance $P_{k+1}^{1}$ on the 1st subsystem is minimized at each time instant.*

**Proof.**  The filtering gain described by (13) is optimal in the sense that minimizes the upper bound of filtering error covariance. Notice that the first, second and third terms on the upper bound (19) are quadratic in $K_{k}^{1}$. The matrix differentiation formula can be applied to Formula (19), and differentiate $tr\left({\bar{P}}_{k+1}^{1}\right)$ with respect to $K_{k}^{1}$. The result is

(25) $$\begin{array}{cc} \frac{\partial \left(tr\left({\bar{P}}_{k+1}^{1}\right)\right)}{\partial \left(K_{k}^{1}\right)} & =-2\alpha_{1}\left(1+\tau_{1}+\tau_{2}\alpha \right)\left({\tilde{A}}_{11}-\alpha_{1}K_{k}^{1}{\tilde{C}}_{11}\right){\bar{P}}_{k}^{1}{\tilde{C}}_{11}^{T}\\ & +2\left(\alpha +\tau_{2}^{-1}\alpha +\tau_{3}^{-1}\alpha \right)\beta^{2}K_{k}^{1}S_{1}S_{1}^{T}+2\alpha_{1}K_{k}^{1}{\tilde{V}}_{1}R_{v}{\left({\tilde{V}}_{1}\right)}^{T}+2\alpha_{2}K_{k}^{1}{\tilde{C}}_{11}\left({\bar{P}}_{k}^{1}+{\hat{\tilde{x}}}_{k}^{1}{\left({\hat{\tilde{x}}}_{k}^{1}\right)}^{T}\right){\tilde{C}}_{11}^{T} \end{array}$$

Now, set the derivative equal to zero and solve for the optimal gain. The following form can be derived:

(26) $$\begin{array}{c} K_{k}^{1}=\alpha_{1}\left(1+\tau_{1}+\tau_{2}\alpha^{2}\right){\tilde{A}}_{11}{\bar{P}}_{k}^{1}{\tilde{C}}_{11}^{T}\\ \times {\left(\alpha_{1}^{2}\left(1+\tau_{1}+\tau_{2}\alpha^{2}\right){\tilde{C}}_{11}{\bar{P}}_{k}^{1}{\tilde{C}}_{11}^{T}+\left(\alpha +\tau_{2}^{-1}+\tau_{3}^{-1}\right)\beta^{2}S_{1}S_{1}^{T}+\alpha_{1}{\tilde{D}}_{v}^{1}R_{v}{\left({\tilde{D}}_{v}^{1}\right)}^{T}+\tilde{X}\right)}^{-1} \end{array}$$

where $\tilde{X}=\alpha_{2}{\tilde{C}}_{11}\left({\bar{P}}_{k}^{1}+{\hat{\tilde{x}}}_{k}^{1}{\left({\hat{\tilde{x}}}_{k}^{1}\right)}^{T}\right){\tilde{C}}_{11}^{T}$. ☐

**Remark** **3.** In the Theorems 1 and 2, the constructed fault-detection filter has been presented for stochastic systems against randomly occurring deception attacks. The available information of the deception attacks has been reflected in our proposed filtering design including α, $\alpha_{1}$, $\alpha_{2}$ and β. In addition, three scalars $\tau_{1}$, $\tau_{2}$ and $\tau_{3}$ have been introduced to enhance the flexibility in our fault-detection filter.

**Remark** **4.** Theorem 2 provides the optimal gain $K_{k}^{1}$ without system faults, which is similar to the discrete-time standard Kalman filter [27]. Consequently, when the 1st subsystem is healthy, the mean-square filtering error is minimized so as to ensure accurate estimation; otherwise, the filtering error will be bigger than the system without fault to achieve the purpose of fault alarming.

### 3.2. Fault-Alarming Strategy

Prior to presenting a novel fault-alarming strategy, a fault indicating signal (i.e., residual), can be generated using the output estimation of the 1st subsystem as follows

(27) $$r_{k}=E\left[{\tilde{y}}_{k}^{1}-{\hat{\tilde{y}}}_{k}^{1}\right]$$

where ${\hat{\tilde{y}}}_{k}^{1}=\alpha_{1}{\tilde{C}}_{11}{\hat{\tilde{x}}}_{k}^{1}$. Once a fault occurs, the residual and the filtering error dynamics of the proposed fault-detection filtering become:

(28) $$\begin{array}{cc} r_{k} & =\alpha_{1}{\tilde{C}}_{11}e_{k}^{1}-\left(\alpha_{k}-\alpha \right){\tilde{C}}_{11}{\tilde{x}}_{k}^{1}+\left(1-\alpha_{k}\right){\tilde{V}}_{1}v_{k}+\alpha_{k}S_{1}\beta_{k}+\left(1-\alpha_{k}\right){\bar{F}}_{1}f_{k}\\ e_{k+1}^{1} & =\left({\tilde{A}}_{11}-\alpha_{1}K_{k}^{1}{\tilde{C}}_{11}\right)e_{k}^{1}+{\tilde{A}}_{12}e_{k}^{2}+{\tilde{W}}_{1}w_{k}-\left(1-\alpha_{k}\right)K_{k}^{1}{\tilde{V}}_{1}v_{k}\\ & -\alpha_{k}K_{k}^{1}S_{1}\beta_{k}-\left(\alpha_{k}-\alpha \right){\tilde{C}}_{11}{\tilde{x}}_{k}^{1}+{\tilde{F}}_{1}f_{k}-\left(1-\alpha_{k}\right)K_{k}^{1}{\bar{F}}_{1}f_{k} \end{array}$$

**Figure 1:**
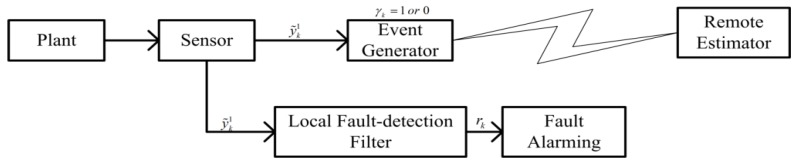
A block diagram of event-triggered fault-alarming strategy.

Now, our event-triggered fault-alarming strategy is presented in Figure 1 and Algorithm 1. As shown in Figure 1, the remote estimation of a system is based on the measurements taken by a battery-powered sensor. The remote estimator receives the measurements through a wireless channel. A copy of remote estimator and event generator (which is also called event-triggered data transmission scheme) determines whether the current measurement information is sent to the remote estimator or not. When an event occurs i.e., $\gamma_{k}=1$, the remote estimator receives the measurement, otherwise $\gamma_{k}=0$. A local estimator runs the fault-detection residual of the above 1st subsystem that we defined. Clearly, if the system is free from faults ($f_{k}\equiv 0$), then $\lim_{t\to \infty }E\left(r_{k}\right)\approx 0$; conversely, if a fault occurs in the system (${\tilde{F}}_{1}f_{k}\neq 0$ and ${\bar{F}}_{1}f_{k}\neq 0$), then $\lim_{t\to \infty }E\left(r_{k}\right)\gg 0$ to achieve fault alarming. Furthermore, a copy of remote estimator, event generator and local estimator are supposed to be integrated in an embedded system which is presented in the experimental verification.

**Algorithm 1** Event-triggered fault detection.**Step 1**: Design a bank of fault-detection filter of the form (13).
**Step 2**: Compute the fault-detection residuals $r_{k}$, and choose a threshold $\delta_{f}$ which can be chosen as small as possible theoretically.
**Step 3**: **If**
$r_{k}<\delta_{f}$, there exists no fault and the corresponding fault-alarming is turned off.
**Step 4**: **If**
$\gamma_{k}=1$, the current measurements can be sent to the remote estimator.
**Step 5**: **else**
$\gamma_{k}=0$, the remote estimator cannot receive the measurements to achieve energy saving.
**Step 6**: **end if**
**Step 7**: **else**
$r_{k}\geq \delta_{f}$, a fault has occured and the corresponding fault alarming is turned on. For the purpose of detecting system fault in the remote estimator, the current sensor measurements is sent to the remote estimator without entering the event-triggered decision.
**Step 8**: **end if** **Step 9**: **end**

As discussed in Algorithm 1, if $r_{k}<\delta_{f}$, it implies that the system is free from fault; and then, the event-triggered data transmission scheme can be utilized to achieve energy conversation. On the other hand, if $r_{k}\geq \delta_{f}$, it is claimed that the system is faulty. The current sensor information is sent to the remote estimator manually without entering event-triggered decision. The delay issue of fault-alarming can be effectively solved, although such strategy may reduce working-life of battery slightly.

## 4. Co-Design Scheme of Fault Estimator and Event-Triggered Generator

After a fault occurs, a fault estimator for subsystem (12) is constructed as follows:

(29) $$\left\{\begin{array}{c} {\hat{\tilde{x}}}_{k+1}^{2}={\tilde{A}}_{22}{\hat{\tilde{x}}}_{k}^{2}+{\tilde{A}}_{21}{\hat{\tilde{x}}}_{k}^{1}+{\hat{F}}_{2}{\hat{f}}_{k}+K_{k}^{2}\left({\tilde{y}}_{p,k}^{2}-{\hat{\tilde{y}}}_{k}^{2}\right)\\ {\hat{f}}_{k+1}={\hat{f}}_{k}+K_{k}^{3}\left({\tilde{y}}_{p,k}^{2}-{\hat{\tilde{y}}}_{k}^{2}\right)\\ {\hat{\tilde{y}}}_{k}^{2}=\alpha_{1}\left({\tilde{C}}_{22}{\hat{\tilde{x}}}_{k}^{2}+F_{2}{\hat{f}}_{k}\right) \end{array}\right.$$

where ${\hat{\tilde{x}}}_{k}^{2}$ is an estimated state and the matrices $K_{k}^{i}$ (i=2 and 3) are estimator’s gains with appropriate dimensions to be designed. Fault estimation signal ${\hat{f}}_{k+1}$ is updated by the estimated information ${\hat{f}}_{k}$ as well as output estimation error $\hat{\tilde{}}y_{k}^{2}$. The previous measurement information ${\tilde{y}}_{p,k}^{2}$ is transmitted from sensor to remote fault estimator module when no new measurement information is transmitted. In the event generator module described in Figure 1, sensor information is not transmitted at each time instant, rather this is done only at the transmission times that are denoted by $k_{s}$ and s∈N. As a result, measurements sent to the remote fault estimator module can be expressed as ${\tilde{y}}_{p,k}^{i}={\tilde{y}}_{k_{s}}^{i}$ (*i* = 1 and 2), $k\in \left[k_{s},\;k_{s+1}\right)$ and k∈N where $k_{s+1}>k_{s}$.

**Remark** **5.** It is worth mentioning that our fault estimator utilizes the previous received measurement information ${\tilde{y}}_{p,k}^{2}$ because of implementation of event generator. As discussed in Algorithm 1, the current measurement information is sent to the remote estimator without entering the event-triggered decision when a fault occurs. However, such a remote fault estimator can ensure that the undetected faults are also estimated if the event condition is triggered.

Denote fault estimation error and the corresponding error covariance as

(30) $$e_{f,k}=f_{d,k}-{\hat{f}}_{k}$$

(31) $$P_{f,k}=E\left[e_{f,k}e_{f,k}^{T}\right]$$

Then, the error dynamics of the 2nd subsystem can be formulated as

(32) $$\begin{array}{cc} e_{k+1}^{2} & ={\tilde{x}}_{k+1}^{2}-{\hat{\tilde{x}}}_{k+1}^{2}\\ & =\left({\tilde{A}}_{22}-\alpha_{1}K_{k}^{2}{\tilde{C}}_{22}\right)e_{k}^{2}+{\tilde{A}}_{21}e_{k}^{1}+\left({\hat{F}}_{2}-\alpha_{1}K_{k}^{2}F_{2}\right)e_{f,k}+{\tilde{W}}_{2}w_{k}-K_{k}^{2}\delta_{k}\\ & -\left(1-\alpha_{k}\right)K_{k}^{2}{\tilde{V}}_{2}v_{k}-\alpha_{k}K_{k}^{2}S_{2}\beta_{k}+\left(\alpha_{k}-\alpha \right)K_{k}^{2}{\tilde{C}}_{22}{\tilde{x}}_{k}^{2}+\left(\alpha_{k}-\alpha \right)K_{k}^{2}F_{2}f_{d,k} \end{array}$$

where $\delta_{k}={\tilde{y}}_{k}^{2}-{\tilde{y}}_{p,k}^{2}$.

(33) $$\begin{array}{cc} e_{f,k+1} & =f_{d,k+1}-{\hat{f}}_{k+1}\\ & =f_{d,k+1}-f_{d,k}+f_{d,k}-{\hat{f}}_{k}-K_{k}^{3}\left({\tilde{y}}_{p,k}^{2}-{\hat{\tilde{y}}}_{k}^{2}\right)\\ & =f_{d,k+1}-f_{d,k}+f_{d,k}-{\hat{f}}_{k}-\alpha_{1}K_{k}^{3}{\tilde{C}}_{22}e_{k}^{2}-\left(1-\alpha_{k}\right)K_{k}^{3}{\tilde{V}}_{2}v_{k}-\alpha_{k}K_{k}^{3}S_{2}\beta_{k}\\ & +\left(\alpha_{k}-\alpha \right)K_{k}^{3}{\tilde{C}}_{22}{\tilde{x}}_{k}^{2}-K_{k}^{3}\delta_{k}+\left(\alpha_{k}-\alpha \right)K_{k}^{3}F_{2}f_{d,k}-\alpha_{1}K_{k}^{3}F_{2}e_{f,k} \end{array}$$

which can be rewritten as

(34) $$\begin{array}{cc} e_{f,k+1} & =\Delta f_{k}+\left(I-\alpha_{1}K_{k}^{3}F_{2}\right)e_{f,k}-\alpha_{1}K_{k}^{3}{\tilde{C}}_{22}e_{k}^{2}-\left(1-\alpha_{k}\right)K_{k}^{3}{\tilde{V}}_{2}v_{k}-\alpha_{k}K_{k}^{3}S_{2}\beta_{k}\\ & +\left(\alpha_{k}-\alpha \right)K_{k}^{3}{\tilde{C}}_{22}{\tilde{x}}_{k}^{2}-K_{k}^{3}\delta_{k}+\left(\alpha_{k}-\alpha \right)K_{k}^{3}F_{2}f_{d,k} \end{array}$$

where $\Delta f_{k}=f_{d,k+1}-f_{d,k}$.

**Remark** **6.** Literature [28,29] assumed that the fault difference item was too small to be neglected, because the sampling interval were supposed to be sufficiently small. However, in many practical cases, faults might generate a great amplitude change of at a certain time, especially when time-varying faults occur. Hence, the paper considers the effect on the estimation performance, which reduces the conservatism of our fault estimator.

Similar to the design of fault-detection filter gain, the gains $K_{k}^{2}$, $K_{k}^{3}$ and the corresponding event-triggered data transmission scheme can be derived such that upper bounds of state and fault estimation error covariance are minimized at each time step. Before proceeding further, the following assumption is made.

**Assumption** **3.** [11] For a small positive constant $b_{d}$, the inequality $∥\left[\begin{array}{c} f_{k+1}\\ d_{k+1} \end{array}\right]-\left[\begin{array}{c} f_{k}\\ d_{k} \end{array}\right]∥\leq b_{d}$ holds, where $∥\cdot ∥$ is the Euclidean norm.

Now, it is ready to give the upper bounds of both the covariance matrix $P_{k}^{2}$ of estimation error and the fault estimation error covariance $P_{f,k}$ in the following theorem.

**Theorem** **3.** *For given positive scalars $\delta_{e}$, $\tau_{j}\left(j=4,\;\ldots ,\;15\right)$ and $\rho_{j}\left(j=1,\;\ldots ,\;12\right)$, the state estimation error covariance $P_{k}^{2}$ and fault estimation error covariance $P_{f,k}$ have the following upper bounds ${\bar{P}}_{k}^{2}$ and ${\bar{P}}_{f,k}$ with initial conditions ${\bar{P}}_{0}^{2}=P_{0}^{2}$ and ${\bar{P}}_{f,0}=P_{f,0}$, respectively, where*

(35) $$\begin{array}{c} {\bar{P}}_{k+1}^{2}=\left(1+\tau_{4}+\tau_{5}+\tau_{6}+\tau_{7}\alpha \right)\left({\tilde{A}}_{22}-\alpha_{1}K_{k}^{2}{\tilde{C}}_{22}\right){\bar{P}}_{k}^{2}{\left({\tilde{A}}_{22}-\alpha_{1}K_{k}^{2}{\tilde{C}}_{22}\right)}^{T}\\ +\left(1+\tau_{4}^{-1}+\tau_{8}+\tau_{9}+\tau_{10}\alpha \right){\tilde{A}}_{21}{\bar{P}}_{k}^{1}{\tilde{A}}_{21}^{T}+\left(1+\tau_{5}^{-1}+\tau_{8}^{-1}+\tau_{11}+\tau_{12}\alpha \right)\left({\hat{F}}_{2}-\alpha_{1}K_{k}^{2}F_{2}\right)\bar{P_{f,k}}{\left({\hat{F}}_{2}-\alpha_{1}K_{k}^{2}F_{2}\right)}^{T}\\ +\left(1+\tau_{6}^{-1}+\tau_{9}^{-1}+\tau_{11}^{-1}+\tau_{13}\alpha \right)\delta_{e}^{2}K_{k}^{2}{\left(K_{k}^{2}\right)}^{T}+\left(\alpha +\tau_{7}^{-1}\alpha +\tau_{10}^{-1}\alpha +\tau_{12}^{-1}\alpha +\tau_{13}^{-1}\alpha \right)K_{k}^{2}S_{2}\beta^{2}S_{2}^{T}{\left(K_{k}^{2}\right)}^{T}\\ +{\tilde{W}}_{2}Q_{w}{\left({\tilde{W}}_{2}\right)}^{T}+\left(1-\alpha \right)K_{k}^{2}{\tilde{V}}_{2}R_{v}{\left({\tilde{V}}_{2}\right)}^{T}{\left(K_{k}^{2}\right)}^{T}+\left(1+\tau_{14}^{-1}\right)\alpha_{2}K_{k}^{2}{\tilde{C}}_{22}\left({\bar{P}}_{k}^{2}+{\hat{\tilde{x}}}_{k}^{2}{\left({\hat{\tilde{x}}}_{k}^{2}\right)}^{T}\right){\tilde{C}}_{22}^{T}{\left(K_{k}^{2}\right)}^{T}\\ +\left(1+\tau_{14}\right)\alpha_{2}K_{k}^{2}F_{2}\left(\left(1+\tau_{15}\right){\bar{P}}_{f,k}+\left(1+\tau_{15}^{-1}\right){\hat{f}}_{k}{\hat{f}}_{k}^{T}\right)F_{2}^{T}{\left(K_{k}^{2}\right)}^{T} \end{array}$$

*and*

(36) $$\begin{array}{c} {\bar{P}}_{f,k+1}=\left(1+\rho_{1}+\rho_{2}\alpha_{1}+\rho_{3}\alpha +\rho_{4}\right)\left(I-\alpha_{1}K_{k}^{3}F_{2}\right){\bar{P}}_{f,k}{\left(I-\alpha_{1}K_{k}^{3}F_{2}\right)}^{T}+\left(1+\rho_{1}^{-1}+\rho_{5}\alpha_{1}+\rho_{6}\alpha +\rho_{7}\right)b_{d}^{2}I\\ +\left(\alpha_{1}^{2}+\rho_{2}^{-1}\alpha_{1}+\rho_{5}^{-1}\alpha_{1}+\rho_{8}\alpha \alpha_{1}+\rho_{9}\alpha_{1}\right)K_{k}^{3}{\tilde{C}}_{22}{\bar{P}}_{k}^{2}{\tilde{C}}_{22}^{T}{\left(K_{k}^{3}\right)}^{T}\\ +\left(\alpha +\rho_{3}^{-1}\alpha +\rho_{6}^{-1}\alpha +\rho_{8}^{-1}\alpha \alpha_{1}+\rho_{10}\alpha \right)\beta^{2}K_{k}^{3}S_{2}S_{2}^{T}{\left(K_{k}^{3}\right)}^{T}\\ +\left(1+\rho_{4}^{-1}+\rho_{7}^{-1}+\rho_{9}^{-1}\alpha_{1}+\rho_{10}^{-1}\alpha \right)\delta_{e}^{2}K_{k}^{3}{\left(K_{k}^{3}\right)}^{T}+\left(1+\rho_{11}\right)\alpha_{2}K_{k}^{3}{\tilde{C}}_{22}\left({\bar{P}}_{k}^{2}+{\hat{\tilde{x}}}_{k}^{2}{\left({\hat{\tilde{x}}}_{k}^{2}\right)}^{T}\right){\tilde{C}}_{22}^{T}{\left(K_{k}^{3}\right)}^{T}\\ +\left(1+\rho_{11}^{-1}\right)\alpha_{2}K_{k}^{3}F_{2}\left(\left(1+\rho_{12}\right){\bar{P}}_{f,k}+\left(1+\rho_{12}^{-1}\right){\hat{f}}_{k}{\hat{f}}_{k}^{T}\right)F_{2}^{T}{\left(K_{k}^{3}\right)}^{T}+\left(1-\alpha \right)K_{k}^{3}{\tilde{V}}_{2}R_{v}{\left({\tilde{V}}_{2}\right)}^{T}{\left(K_{k}^{3}\right)}^{T} \end{array}$$

*Furthermore, the event condition satisfies that*

(37) $$∥{\tilde{y}}_{k}^{2}-{\tilde{y}}_{p,k}^{2}∥\leq \delta_{e}$$

**Proof.**  First, recall from the definition of state estimation error covariance (16), $P_{k}^{2}$ can be calculated as follows

$$\begin{array}{cc} P_{k+1}^{2} & =E\left[\left({\tilde{x}}_{k}^{2}-{\hat{\tilde{x}}}_{k}^{2}\right){\left({\tilde{x}}_{k}^{2}-{\hat{\tilde{x}}}_{k}^{2}\right)}^{T}\right]\\ & =E\left[\left({\tilde{A}}_{22}-\alpha_{1}K_{k}^{2}{\tilde{C}}_{22}\right)\right.e_{k}^{2}+{\tilde{A}}_{21}e_{k}^{1}+\left({\hat{F}}_{2}-\alpha_{1}K_{k}^{2}F_{2}\right)e_{f,k}+{\tilde{W}}_{2}w_{k}-K_{k}^{2}\delta_{k}\\ & \left.-\left(1-\alpha_{k}\right)K_{k}^{2}{\tilde{V}}_{2}v_{k}-\alpha_{k}K_{k}^{2}S_{2}\beta_{k}+\left(\alpha_{k}-\alpha \right)K_{k}^{2}{\tilde{C}}_{22}{\tilde{x}}_{k}^{2}+\left(\alpha_{k}-\alpha \right)K_{k}^{2}F_{2}f_{d,k}\right]\\ & \times \left[\left({\tilde{A}}_{22}-\alpha_{1}K_{k}^{2}{\tilde{C}}_{22}\right)\right.e_{k}^{2}+{\tilde{A}}_{21}e_{k}^{1}+\left({\hat{F}}_{2}-\alpha_{1}K_{k}^{2}F_{2}\right)e_{f,k}+{\tilde{W}}_{2}w_{k}-K_{k}^{2}\delta_{k}\\ & {\left.-\left(1-\alpha_{k}\right)K_{k}^{2}{\tilde{V}}_{2}v_{k}-\alpha_{k}K_{k}^{2}S_{2}\beta_{k}+\left(\alpha_{k}-\alpha \right)K_{k}^{2}{\tilde{C}}_{22}{\tilde{x}}_{k}^{2}+\left(\alpha_{k}-\alpha \right)K_{k}^{2}F_{2}f_{d,k}\right]}^{T}\\ & =\left({\tilde{A}}_{22}-\alpha_{1}K_{k}^{2}{\tilde{C}}_{22}\right)P_{k}^{2}{\left({\tilde{A}}_{22}-\alpha_{1}K_{k}^{2}{\tilde{C}}_{22}\right)}^{T}+{\tilde{A}}_{21}P_{k}^{1}{\tilde{A}}_{21}^{T}\\ & +\left({\hat{F}}_{2}-\alpha_{1}K_{k}^{2}F_{2}\right)P_{f,k}{\left({\hat{F}}_{2}-\alpha_{1}K_{k}^{2}F_{2}\right)}^{T}+{\tilde{W}}_{2}Q_{w}{\left({\tilde{W}}_{2}\right)}^{T}+K_{k}^{2}\delta_{k}\delta_{k}^{T}{\left(K_{k}^{2}\right)}^{T}\\ & +\left(1-\alpha \right)K_{k}^{2}{\tilde{V}}_{2}R_{v}{\left({\tilde{V}}_{2}\right)}^{T}{\left(K_{k}^{2}\right)}^{T}+\alpha K_{k}^{2}S_{2}\beta_{k}\beta_{k}^{T}S_{2}^{T}{\left(K_{k}^{2}\right)}^{T} \end{array}$$

(38) $$\begin{array}{c} +\left({\tilde{A}}_{22}-\alpha_{1}K_{k}^{2}{\tilde{C}}_{22}\right)E\left[e_{k}^{2}{\left(e_{k}^{1}\right)}^{T}\right]{\tilde{A}}_{21}^{T}+{\tilde{A}}_{21}E\left[e_{k}^{1}{\left(e_{k}^{2}\right)}^{T}\right]{\left({\tilde{A}}_{22}-\alpha_{1}K_{k}^{2}{\tilde{C}}_{22}\right)}^{T}\\ +\left({\tilde{A}}_{22}-\alpha_{1}K_{k}^{2}{\tilde{C}}_{22}\right)E\left[e_{k}^{2}e_{f,k}^{T}\right]{\left({\hat{F}}_{2}-\alpha_{1}K_{k}^{2}F_{2}\right)}^{T}+\left({\hat{F}}_{2}-\alpha_{1}K_{k}^{2}F_{2}\right)E\left[e_{f,k}{\left(e_{k}^{2}\right)}^{T}\right]\left({\tilde{A}}_{22}-\alpha_{1}K_{k}^{2}{\tilde{C}}_{22}\right)\\ -\left({\tilde{A}}_{22}-\alpha_{1}K_{k}^{2}{\tilde{C}}_{22}\right)E\left[e_{k}^{2}\delta_{k}^{T}\right]{\left(K_{k}^{2}\right)}^{T}-K_{k}^{2}E\left[\delta_{k}{\left(e_{k}^{2}\right)}^{T}\right]{\left({\tilde{A}}_{22}-\alpha_{1}K_{k}^{2}{\tilde{C}}_{22}\right)}^{T}\\ -\alpha \left({\tilde{A}}_{22}-\alpha_{1}K_{k}^{2}{\tilde{C}}_{22}\right)E\left[e_{k}^{2}\beta_{k}^{T}\right]S_{2}^{T}{\left(K_{k}^{2}\right)}^{T}-\alpha K_{k}^{2}S_{2}E\left[\beta_{k}{\left(e_{k}^{2}\right)}^{T}\right]{\left({\tilde{A}}_{22}-\alpha_{1}K_{k}^{2}{\tilde{C}}_{22}\right)}^{T}\\ +{\tilde{A}}_{21}E\left[e_{k}^{1}e_{f,k}^{T}\right]{\left({\hat{F}}_{2}-\alpha_{1}K_{k}^{2}F_{2}\right)}^{T}+\left({\hat{F}}_{2}-\alpha_{1}K_{k}^{2}F_{2}\right)E\left[e_{f,k}{\left(e_{k}^{1}\right)}^{T}\right]{\tilde{A}}_{21}^{T}-{\tilde{A}}_{21}E\left[e_{k}^{1}\delta_{k}^{T}\right]{\left(K_{k}^{2}\right)}^{T}\\ -K_{k}^{2}E\left[\delta_{k}{\left(e_{k}^{1}\right)}^{T}\right]{\tilde{A}}_{21}^{T}-\alpha {\tilde{A}}_{21}E\left[e_{k}^{1}\beta_{k}^{T}\right]S_{2}^{T}{\left(K_{k}^{2}\right)}^{T}-\alpha K_{k}^{2}S_{2}E\left[\beta_{k}{\left(e_{k}^{1}\right)}^{T}\right]{\tilde{A}}_{21}^{T}\\ -\left({\hat{F}}_{2}-\alpha_{1}K_{k}^{2}F_{2}\right)E\left[e_{f,k}\delta_{k}^{T}\right]{\left(K_{k}^{2}\right)}^{T}-K_{k}^{2}E\left[\delta_{k}e_{f,k}^{T}\right]{\left({\hat{F}}_{2}-\alpha_{1}K_{k}^{2}F_{2}\right)}^{T}\\ -\alpha \left({\hat{F}}_{2}-\alpha_{1}K_{k}^{2}F_{2}\right)E\left[e_{f,k}\beta_{k}^{T}\right]S_{2}^{T}{\left(K_{k}^{2}\right)}^{T}-\alpha K_{k}^{2}S_{2}E\left[\beta_{k}e_{f,k}^{T}\right]{\left({\hat{F}}^{2}\right)}^{T}+\alpha K_{k}^{2}E\left[\delta_{k}\beta_{k}^{T}\right]S_{2}^{T}{\left(K_{k}^{2}\right)}^{T}\\ +\alpha K_{k}^{2}S_{2}E\left[\beta_{k}\delta_{k}^{T}\right]{\left(K_{k}^{2}\right)}^{T}+\alpha_{2}K_{k}^{2}{\tilde{C}}_{22}E\left[{\tilde{x}}_{k}^{2}{\left({\tilde{x}}_{k}^{2}\right)}^{T}\right]{\tilde{C}}_{22}^{T}{\left(K_{k}^{2}\right)}^{T}+\alpha_{2}K_{k}^{2}F_{2}E\left[f_{d,k}{\left(f_{d,k}\right)}^{T}\right]F_{2}^{T}{\left(K_{k}^{2}\right)}^{T}\\ +\alpha_{2}K_{k}^{2}{\tilde{C}}_{22}E\left[{\tilde{x}}_{k}^{2}{\left(f_{d,k}\right)}^{T}\right]F_{2}^{T}{\left(K_{k}^{2}\right)}^{T}+\alpha_{2}K_{k}^{2}F_{2}E\left[f_{d,k}{\left({\tilde{x}}_{k}^{2}\right)}^{T}\right]{\tilde{C}}_{22}^{T}{\left(K_{k}^{2}\right)}^{T} \end{array}$$

By using the result of Lemma 1, the above equation can be simplified as

(39) $$\begin{array}{c} P_{k+1}^{2}\leq \left(1+\tau_{4}+\tau_{5}+\tau_{6}+\tau_{7}\alpha \right)\left({\tilde{A}}_{22}-\alpha_{1}K_{k}^{2}{\tilde{C}}_{22}\right)P_{k}^{2}{\left({\tilde{A}}_{22}-\alpha_{1}K_{k}^{2}{\tilde{C}}_{22}\right)}^{T}\\ +\left(1+\tau_{4}^{-1}+\tau_{8}+\tau_{9}+\tau_{10}\alpha \right){\tilde{A}}_{21}P_{k}^{1}{\tilde{A}}_{21}^{T}+\left(1+\tau_{5}^{-1}+\tau_{8}^{-1}+\tau_{11}+\tau_{12}\alpha \right)\left({\hat{F}}_{2}-\alpha_{1}K_{k}^{2}F_{2}\right)P_{f,k}{\left({\hat{F}}_{2}-\alpha_{1}K_{k}^{2}F_{2}\right)}^{T}\\ +\left(1+\tau_{6}^{-1}+\tau_{9}^{-1}+\tau_{11}^{-1}+\tau_{13}\alpha \right)K_{k}^{2}\delta_{k}\delta_{k}^{T}{\left(K_{k}^{2}\right)}^{T}+\left(\alpha +\tau_{7}^{-1}\alpha +\tau_{10}^{-1}\alpha +\tau_{12}^{-1}\alpha +\tau_{13}^{-1}\alpha \right)K_{k}^{2}S_{2}\beta_{k}\beta_{k}^{T}S_{2}^{T}{\left(K_{k}^{2}\right)}^{T}\\ +{\tilde{W}}_{2}Q_{w}{\left({\tilde{W}}_{2}\right)}^{T}+\left(1-\alpha \right)K_{k}^{2}{\tilde{V}}_{2}R_{v}{\left({\tilde{V}}_{2}\right)}^{T}{\left(K_{k}^{2}\right)}^{T}+\left(1+\tau_{14}^{-1}\right)\alpha_{2}K_{k}^{2}{\tilde{C}}_{22}E\left[{\tilde{x}}_{k}^{2}{\left({\tilde{x}}_{k}^{2}\right)}^{T}\right]{\tilde{C}}_{22}^{T}{\left(K_{k}^{2}\right)}^{T}\\ +\left(1+\tau_{14}\right)\alpha_{2}K_{k}^{2}F_{2}\left(\left(1+\tau_{15}\right)P_{f,k}+\left(1+\tau_{15}^{-1}\right){\hat{f}}_{k}{\hat{f}}_{k}^{T}\right)F_{2}^{T}{\left(K_{k}^{2}\right)}^{T} \end{array}$$

Considering that $E\left[{\tilde{x}}_{k}^{2}{\left({\tilde{x}}_{k}^{2}\right)}^{T}\right]=P_{k}^{2}+{\hat{\tilde{x}}}_{k}^{2}{\left({\hat{\tilde{x}}}_{k}^{2}\right)}^{T}$ and $∥\beta_{k}∥\leq \beta$, (39) can be further reduced as

(40) $$\begin{array}{c} P_{k+1}^{2}\leq \left(1+\tau_{4}+\tau_{5}+\tau_{6}+\tau_{7}\alpha \right)\left({\tilde{A}}_{22}-\alpha_{1}K_{k}^{2}{\tilde{C}}_{22}\right)P_{k}^{2}{\left({\tilde{A}}_{22}-\alpha_{1}K_{k}^{2}{\tilde{C}}_{22}\right)}^{T}\\ +\left(1+\tau_{4}^{-1}+\tau_{8}+\tau_{9}+\tau_{10}\alpha \right){\tilde{A}}_{21}P_{k}^{1}{\tilde{A}}_{21}^{T}+\left(1+\tau_{5}^{-1}+\tau_{8}^{-1}+\tau_{11}+\tau_{12}\alpha \right)\left({\hat{F}}_{2}-\alpha_{1}K_{k}^{2}F_{2}\right)P_{f,k}\left({\hat{F}}_{2}-\alpha_{1}K_{k}^{2}F_{2}\right)\\ +\left(1+\tau_{6}^{-1}+\tau_{9}^{-1}+\tau_{11}^{-1}+\tau_{13}\alpha \right)K_{k}^{2}\delta_{k}\delta_{k}^{T}{\left(K_{k}^{2}\right)}^{T}+\left(\alpha +\tau_{7}^{-1}\alpha +\tau_{10}^{-1}\alpha +\tau_{12}^{-1}\alpha +\tau_{13}^{-1}\alpha \right)K_{k}^{2}S_{2}\beta^{2}S_{2}^{T}{\left(K_{k}^{2}\right)}^{T}\\ +{\tilde{W}}_{2}Q_{w}{\left({\tilde{W}}_{2}\right)}^{T}+\left(1-\alpha \right)K_{k}^{2}{\tilde{V}}_{2}R_{v}{\left({\tilde{V}}_{2}\right)}^{T}{\left(K_{k}^{2}\right)}^{T}+\left(1+\tau_{14}^{-1}\right)\alpha_{2}K_{k}^{2}{\tilde{C}}_{22}\left(P_{k}^{2}+{\hat{\tilde{x}}}_{k}^{2}{\left({\hat{\tilde{x}}}_{k}^{2}\right)}^{T}\right){\tilde{C}}_{22}^{T}{\left(K_{k}^{2}\right)}^{T}\\ +\left(1+\tau_{14}\right)\alpha_{2}K_{k}^{2}F_{2}\left(\left(1+\tau_{15}\right)P_{f,k}+\left(1+\tau_{15}^{-1}\right){\hat{f}}_{k}{\hat{f}}_{k}^{T}\right)F_{2}^{T}{\left(K_{k}^{2}\right)}^{T} \end{array}$$

If the event condition is satisfied, i.e., $∥{\tilde{y}}_{k}^{2}-{\tilde{y}}_{p,k}^{2}∥\leq \delta_{e}$, we have

(41) $$\begin{array}{c} P_{k+1}^{2}\leq \left(1+\tau_{4}+\tau_{5}+\tau_{6}+\tau_{7}\alpha \right)\left({\tilde{A}}_{22}-\alpha_{1}K_{k}^{2}{\tilde{C}}_{22}\right)P_{k}^{2}{\left({\tilde{A}}_{22}-\alpha_{1}K_{k}^{2}{\tilde{C}}_{22}\right)}^{T}\\ +\left(1+\tau_{4}^{-1}+\tau_{8}+\tau_{9}+\tau_{10}\alpha \right){\tilde{A}}_{21}P_{k}^{1}{\tilde{A}}_{21}^{T}+\left(1+\tau_{5}^{-1}+\tau_{8}^{-1}+\tau_{11}+\tau_{12}\alpha \right)\left({\hat{F}}_{2}-\alpha_{1}K_{k}^{2}F_{2}\right)P_{f,k}{\left({\hat{F}}_{2}-\alpha_{1}K_{k}^{2}F_{2}\right)}^{T}\\ +\left(1+\tau_{6}^{-1}+\tau_{9}^{-1}+\tau_{11}^{-1}+\tau_{13}\alpha \right)\delta_{e}^{2}K_{k}^{2}{\left(K_{k}^{2}\right)}^{T}+\left(\alpha +\tau_{7}^{-1}\alpha +\tau_{10}^{-1}\alpha +\tau_{12}^{-1}\alpha +\tau_{13}^{-1}\alpha \right)K_{k}^{2}S_{2}\beta^{2}S_{2}^{T}{\left(K_{k}^{2}\right)}^{T}\\ +{\tilde{W}}_{2}Q_{w}{\left({\tilde{W}}_{2}\right)}^{T}+\left(1-\alpha \right)K_{k}^{2}{\tilde{V}}_{2}R_{v}{\left({\tilde{V}}_{2}\right)}^{T}{\left(K_{k}^{2}\right)}^{T}+\left(1+\tau_{14}^{-1}\right)\alpha_{2}K_{k}^{2}{\tilde{C}}_{22}\left(P_{k}^{2}+{\hat{\tilde{x}}}_{k}^{2}{\left({\hat{\tilde{x}}}_{k}^{2}\right)}^{T}\right){\tilde{C}}_{22}^{T}{\left(K_{k}^{2}\right)}^{T}\\ +\left(1+\tau_{14}\right)\alpha_{2}K_{k}^{2}F_{2}\left(\left(1+\tau_{15}\right)P_{f,k}+\left(1+\tau_{15}^{-1}\right){\hat{f}}_{k}{\hat{f}}_{k}^{T}\right)F_{2}^{T}{\left(K_{k}^{2}\right)}^{T} \end{array}$$

Next, let us calculate the error covariance of fault estimation as follows

(42) $$\begin{array}{c} P_{f,k+1}=E\left[e_{f,k+1}e_{f,k+1}^{T}\right]\\ =E\left[\Delta f_{k}+\left(I-\alpha_{1}K_{k}^{3}F_{2}\right)e_{f,k}-\alpha_{1}K_{k}^{3}{\tilde{C}}_{22}e_{k}^{2}-\left(1-\alpha_{k}\right)K_{k}^{3}{\tilde{V}}_{2}v_{k}\right.\\ \left.-\alpha_{k}K_{k}^{3}S_{2}\beta_{k}-K_{k}^{3}\delta_{k}+\left(\alpha_{k}-\alpha \right)K_{k}^{3}{\tilde{C}}_{22}{\tilde{x}}_{k}^{2}+\left(\alpha_{k}-\alpha \right)K_{k}^{3}F_{2}f_{d,k}\right]\\ \times \left[\Delta f_{k}+\left(I-\alpha_{1}K_{k}^{3}F_{2}\right)e_{f,k}-\alpha_{1}K_{k}^{3}{\tilde{C}}_{22}e_{k}^{2}-\left(1-\alpha_{k}\right)K_{k}^{3}{\tilde{V}}_{2}v_{k}\right.\\ {\left.-\alpha_{k}K_{k}^{3}S_{2}\beta_{k}-K_{k}^{3}\delta_{k}+\left(\alpha_{k}-\alpha \right)K_{k}^{3}{\tilde{C}}_{22}{\tilde{x}}_{k}^{2}+\left(\alpha_{k}-\alpha \right)K_{k}^{3}F_{2}f_{d,k}\right]}^{T}\\ =\left(I-\alpha_{1}K_{k}^{3}F_{2}\right)P_{f,k}{\left(I-\alpha_{1}K_{k}^{3}F_{2}\right)}^{T}+\Delta f_{k}\Delta f_{k}^{T}+\alpha_{1}^{2}K_{k}^{3}{\tilde{C}}_{22}P_{k}^{2}{\tilde{C}}_{22}^{T}{\left(K_{k}^{3}\right)}^{T}+\left(1-\alpha \right)K_{k}^{3}{\tilde{V}}_{2}R_{v}{\left({\tilde{V}}_{2}\right)}^{T}{\left(K_{k}^{3}\right)}^{T}\\ +\alpha K_{k}^{3}S_{2}\beta_{k}\beta_{k}^{T}S_{2}^{T}{\left(K_{k}^{3}\right)}^{T}+K_{k}^{3}\delta_{k}\delta_{k}^{T}{\left(K_{k}^{3}\right)}^{T}+\alpha_{2}K_{k}^{3}{\tilde{C}}_{22}E\left[{\tilde{x}}_{k}^{2}{\left({\tilde{x}}_{k}^{2}\right)}^{T}\right]{\tilde{C}}_{22}^{T}{\left(K_{k}^{3}\right)}^{T}\\ +\alpha_{2}K_{k}^{3}F_{2}E\left[f_{d,k}f_{d,k}^{T}\right]F_{2}^{T}{\left(K_{k}^{3}\right)}^{T}+\alpha_{2}K_{k}^{3}{\tilde{C}}_{22}E\left[{\tilde{x}}_{k}^{2}{\left(f_{d,k}\right)}^{T}\right]F_{2}^{T}{\left(K_{k}^{3}\right)}^{T}+\alpha_{2}K_{k}^{3}F_{2}E\left[f_{d,k}{\left({\tilde{x}}_{k}^{2}\right)}^{T}\right]{\tilde{C}}_{22}^{T}{\left(K_{k}^{3}\right)}^{T}\\ +E\left[\Delta f_{k}e_{f,k}^{T}{\left(I-\alpha_{1}K_{k}^{3}F_{2}\right)}^{T}\right]+E\left[\left(I-\alpha_{1}K_{k}^{3}F_{2}\right)e_{f,k}\Delta f_{k}^{T}\right]-\alpha_{1}E\left[\Delta f_{k}{\left(e_{k}^{2}\right)}^{T}\right]{\tilde{C}}_{22}^{T}{\left(K_{k}^{3}\right)}^{T}\\ -\alpha_{1}K_{k}^{3}{\tilde{C}}_{22}E\left[e_{k}^{2}\Delta f_{k}^{T}\right]-\alpha E\left[\Delta f_{k}\beta_{k}^{T}\right]S_{2}^{T}{\left(K_{k}^{3}\right)}^{T}-\alpha K_{k}^{3}S_{2}E\left[\beta_{k}\Delta f_{k}^{T}\right]-E\left[\Delta f_{k}\delta_{k}^{T}\right]{\left(K_{k}^{3}\right)}^{T}\\ -\alpha_{1}\left(I-\alpha_{1}K_{k}^{3}F_{2}\right)E\left[e_{f,k}{\left(e_{k}^{2}\right)}^{T}\right]{\tilde{C}}_{22}^{T}{\left(K_{k}^{3}\right)}^{T}-\alpha_{1}K_{k}^{3}{\tilde{C}}_{22}E\left[e_{k}^{2}e_{f,k}^{T}\right]{\left(I-\alpha_{1}K_{k}^{3}F_{2}\right)}^{T}\\ -\alpha \left(I-\alpha_{1}K_{k}^{3}F_{2}\right)E\left[e_{f,k}\beta_{k}^{T}\right]S_{2}^{T}{\left(K_{k}^{3}\right)}^{T}-\alpha K_{k}^{3}S_{2}E\left[\beta_{k}e_{f,k}^{T}\right]{\left(I-\alpha_{1}K_{k}^{3}F_{2}\right)}^{T}\\ -\left(I-\alpha_{1}K_{k}^{3}F_{2}\right)E\left[e_{f,k}\delta_{k}^{T}\right]{\left(K_{k}^{3}\right)}^{T}-K_{k}^{3}E\left[\delta_{k}e_{f,k}^{T}\right]{\left(I-\alpha_{1}K_{k}^{3}F_{2}\right)}^{T}+\alpha_{1}\alpha K_{k}^{3}{\tilde{C}}_{22}E\left[e_{k}^{2}\beta_{k}^{T}\right]S_{2}^{T}{\left(K_{k}^{3}\right)}^{T}\\ +\alpha_{1}\alpha K_{k}^{3}S_{2}E\left[\beta_{k}{\left(e_{k}^{2}\right)}^{T}\right]{\tilde{C}}_{22}^{T}{\left(K_{k}^{3}\right)}^{T}+\alpha_{1}K_{k}^{3}{\tilde{C}}_{22}E\left[e_{k}^{2}\delta_{k}^{T}\right]{\left(K_{k}^{3}\right)}^{T}-K_{k}^{3}E\left[\delta_{k}\Delta f_{k}^{T}\right]\\ +\alpha_{1}K_{k}^{3}E\left[\delta_{k}{\left(e_{k}^{2}\right)}^{T}\right]{\tilde{C}}_{22}^{T}{\left(K_{k}^{3}\right)}^{T}+\alpha K_{k}^{3}S_{2}E\left[\beta_{k}\delta_{k}^{T}\right]{\left(K_{k}^{3}\right)}^{T}+\alpha K_{k}^{3}E\left[\delta_{k}\beta_{k}^{T}\right]S_{2}^{T}{\left(K_{k}^{3}\right)}^{T} \end{array}$$

Similar to the derivations of (39), (40) and (41), the upper bound of $P_{f,k+1}$ can be given by

(43) $$\begin{array}{c} P_{f,k+1}\leq \left(1+\rho_{1}+\rho_{2}\alpha_{1}+\rho_{3}\alpha +\rho_{4}\right)\left(I-\alpha_{1}K_{k}^{3}F_{2}\right)P_{f,k}{\left(I-\alpha_{1}K_{k}^{3}F_{2}\right)}^{T}+\left(1+\rho_{1}^{-1}+\rho_{5}\alpha_{1}+\rho_{6}\alpha +\rho_{7}\right)b_{d}^{2}I\\ +\left(\alpha_{1}^{2}+\rho_{2}^{-1}\alpha_{1}+\rho_{5}^{-1}\alpha_{1}+\rho_{8}\alpha \alpha_{1}+\rho_{9}\alpha_{1}\right)K_{k}^{3}{\tilde{C}}_{22}P_{k}^{2}{\tilde{C}}_{22}^{T}{\left(K_{k}^{3}\right)}^{T}\\ +\left(\alpha +\rho_{3}^{-1}\alpha +\rho_{6}^{-1}\alpha +\rho_{8}^{-1}\alpha \alpha_{1}+\rho_{10}\alpha \right)\beta^{2}K_{k}^{3}S_{2}S_{2}^{T}{\left(K_{k}^{3}\right)}^{T}\\ +\left(1+\rho_{4}^{-1}+\rho_{7}^{-1}+\rho_{9}^{-1}\alpha_{1}+\rho_{10}^{-1}\alpha \right)\delta_{e}^{2}K_{k}^{3}{\left(K_{k}^{3}\right)}^{T}+\left(1+\rho_{11}\right)\alpha_{2}K_{k}^{3}{\tilde{C}}_{22}\left(P_{k}^{2}+{\hat{\tilde{x}}}_{k}^{2}{\left({\hat{\tilde{x}}}_{k}^{2}\right)}^{T}\right){\tilde{C}}_{22}^{T}{\left(K_{k}^{3}\right)}^{T}\\ +\left(1+\rho_{11}^{-1}\right)\alpha_{2}K_{k}^{3}F_{2}\left(\left(1+\rho_{12}\right)e_{f,k}e_{f,k}^{T}+\left(1+\rho_{12}^{-1}\right){\hat{f}}_{k}{\hat{f}}_{k}^{T}\right)F_{2}^{T}{\left(K_{k}^{3}\right)}^{T}+\left(1-\alpha \right)K_{k}^{3}{\tilde{V}}_{2}R_{v}{\left({\tilde{V}}_{2}\right)}^{T}{\left(K_{k}^{3}\right)}^{T} \end{array}$$

where $\Delta f_{k}\Delta f_{k}^{T}\leq b_{d}^{2}I$. Subsequently, by employing the mathematical induction approach, it is not difficult to verify that

(44) $$P_{f,k+1}\leq {\bar{P}}_{f,k+1}\;\mathrm{and}\;P_{k+1}^{2}\leq {\bar{P}}_{k+1}^{2}$$

which completes the proof of upper bounds of state and fault estimation error covariance matrices. ☐

In what follows, the explicit forms of the fault estimator gains will be given via minimizing the obtained upper bounds ${\bar{P}}_{f,k+1}$ and ${\bar{P}}_{k+1}^{2}$ at each time step.

**Theorem** **4.** *For the addressed subsystem (12) suffering from deception attacks, the gains of the proposed fault estimator (29) are given by*

(45) $$\begin{array}{cc} K_{k}^{2} & =\left(\alpha_{1}{\tilde{\tau }}_{1}{\tilde{A}}_{22}{\bar{P}}_{k}^{2}{\tilde{C}}_{22}^{T}+\alpha_{1}{\tilde{\tau }}_{2}{\hat{F}}_{2}{\bar{P}}_{f,k}F_{2}^{T}\right)\left(\alpha_{1}^{2}{\tilde{\tau }}_{1}{\tilde{C}}_{22}{\bar{P}}_{k}^{2}{\tilde{C}}_{22}^{T}+\alpha_{1}^{2}{\tilde{\tau }}_{2}F_{2}{\bar{P}}_{f,k}F_{2}^{T}+{\tilde{\tau }}_{3}\delta_{e}^{2}I+{\tilde{\tau }}_{4}S_{2}\beta^{2}S_{2}^{T}\right.\\ & {\left.+\left(1-\alpha \right){\tilde{V}}_{2}R_{v}{\left({\tilde{V}}_{2}\right)}^{T}+\left(1+\tau_{14}\right)\alpha_{2}{\tilde{M}}_{1}+\left(1+\tau_{14}^{-1}\right)\alpha_{2}{\tilde{M}}_{2}\right)}^{-1} \end{array}$$

(46) $$\begin{array}{cc} K_{k}^{3} & ={\tilde{\rho }}_{1}\alpha_{1}{\bar{P}}_{f,k}F_{2}^{T}\left({\tilde{\rho }}_{1}\alpha_{1}^{2}F_{2}{\bar{P}}_{f,k}F_{2}^{T}+{\tilde{\rho }}_{2}{\tilde{C}}_{22}{\bar{P}}_{k}^{2}{\tilde{C}}_{22}^{T}+{\tilde{\rho }}_{3}S_{2}\beta^{2}S_{2}^{T}+{\tilde{\rho }}_{4}\delta_{e}^{2}I\right.\\ & +\left(1+\rho_{11}\right)\alpha_{2}{\tilde{M}}_{2}{\left.+\left(1+\rho_{11}^{-1}\right)\alpha_{2}{\tilde{M}}_{3}+\left(1-\alpha \right){\tilde{V}}_{2}R_{v}{\left({\tilde{V}}_{2}\right)}^{T}\right)}^{-1} \end{array}$$

*where ${\tilde{\tau }}_{1}=1+\tau_{4}+\tau_{5}+\tau_{6}+\tau_{7}\alpha$, ${\tilde{\tau }}_{2}=1+\tau_{5}^{-1}+\tau_{8}^{-1}+\tau_{11}+\tau_{12}\alpha$, ${\tilde{\tau }}_{3}=1+\tau_{6}^{-1}+\tau_{9}^{-1}+\tau_{11}^{-1}+\tau_{13}\alpha$, ${\tilde{\tau }}_{4}=\left(1+\tau_{13}^{-1}+\tau_{7}^{-1}+\tau_{10}^{-1}+\tau_{12}^{-1}\right)\alpha$, ${\tilde{M}}_{1}=F_{2}\left(\left(1+\tau_{15}\right){\bar{P}}_{f,k}+\left(1+\tau_{15}^{-1}\right){\hat{f}}_{k}{\hat{f}}_{k}^{T}\right)F_{2}^{T}$, ${\tilde{M}}_{2}={\tilde{C}}_{22}\left({\bar{P}}_{k}^{2}+{\hat{\tilde{x}}}_{k}^{2}{\left({\hat{\tilde{x}}}_{k}^{2}\right)}^{T}\right){\tilde{C}}_{22}^{T}$, ${\tilde{\rho }}_{1}=1+\rho_{1}+\rho_{2}\alpha_{1}+\rho_{3}\alpha +\rho_{4}$, ${\tilde{\rho }}_{2}=\alpha_{1}^{2}+\rho_{2}^{-1}\alpha_{1}+\rho_{5}^{-1}\alpha_{1}+\rho_{8}\alpha \alpha_{1}+\rho_{9}\alpha_{1}$, ${\tilde{\rho }}_{3}=\alpha +\rho_{3}^{-1}\alpha +\rho_{6}^{-1}\alpha +\rho_{8}^{-1}\alpha \alpha_{1}+\rho_{10}\alpha$, ${\tilde{\rho }}_{4}=1+\rho_{4}^{-1}+\rho_{7}^{-1}+\rho_{9}^{-1}\alpha_{1}+\rho_{10}^{-1}\alpha$ and ${\tilde{M}}_{3}=F_{2}\left(\left(1+\rho_{12}\right){\bar{P}}_{f,k}+\left(1+\rho_{12}^{-1}\right){\hat{f}}_{k}{\hat{f}}_{k}^{T}\right)F_{2}^{T}$.*

With the aid of estimator gains (45) and (46), the obtained upper bounds ${\bar{P}}_{k+1}^{2}$ and ${\bar{P}}_{f,k+1}$ of the state and fault estimation error-covariance matrices in (38) and (42) can separately be minimized at each time step.

**Proof.**  According to Theorem 3, the design of gains $K_{k}^{2}$ and $K_{k}^{3}$ needs to be minimized by $tr\left({\bar{P}}_{k+1}^{2}\right)$ and $tr\left({\bar{P}}_{f,k+1}\right)$, respectively. For this purpose, taking the derivatives of $tr\left({\bar{P}}_{k+1}^{2}\right)$ and $tr\left({\bar{P}}_{f,k+1}\right)$ with respect to $K_{k}^{2}$ and $K_{k}^{3}$, respectively; and then, getting the derivatives be zero, we obtain

(47) $$\begin{array}{cc} \frac{\partial \left(tr\left({\bar{P}}_{k+1}^{2}\right)\right)}{\partial \left(K_{k}^{2}\right)} & =-2\alpha_{1}{\tilde{\tau }}_{1}\left({\tilde{A}}_{22}-\alpha_{1}K_{k}^{2}{\tilde{C}}_{22}\right){\bar{P}}_{k}^{2}{\tilde{C}}_{22}^{T}-2\alpha_{1}{\tilde{\tau }}_{2}\left({\hat{F}}_{2}-\alpha_{1}K_{k}^{2}F_{2}\right){\bar{P}}_{f,k}F_{2}^{T}+2{\tilde{\tau }}_{3}\delta_{e}^{2}K_{k}^{2}+2{\tilde{\tau }}_{4}K_{k}^{2}S_{2}\beta^{2}S_{2}^{T}\\ & +2\left(1-\alpha \right)K_{k}^{2}{\tilde{V}}_{2}R_{v}{\left({\tilde{V}}_{2}\right)}^{T}+2\left(1+\tau_{14}\right)\alpha_{2}K_{k}^{2}{\tilde{M}}_{1}+2\left(1+\tau_{14}^{-1}\right)\alpha_{2}K_{k}^{2}{\tilde{M}}_{2}=0 \end{array}$$

where ${\tilde{\tau }}_{1}=1+\tau_{4}+\tau_{5}+\tau_{6}+\tau_{7}\alpha$, ${\tilde{\tau }}_{2}=1+\tau_{5}^{-1}+\tau_{8}^{-1}+\tau_{11}+\tau_{12}\alpha$, ${\tilde{\tau }}_{3}=1+\tau_{6}^{-1}+\tau_{9}^{-1}+\tau_{11}^{-1}+\tau_{13}\alpha$, ${\tilde{\tau }}_{4}=\left(1+\tau_{13}^{-1}+\tau_{7}^{-1}+\tau_{10}^{-1}+\tau_{12}^{-1}\right)\alpha$, ${\tilde{M}}_{1}=F_{2}\left(\left(1+\tau_{15}\right){\bar{P}}_{f,k}+\left(1+\tau_{15}^{-1}\right){\hat{f}}_{k}{\hat{f}}_{k}^{T}\right)F_{2}^{T}$ and ${\tilde{M}}_{2}={\tilde{C}}_{22}\left({\bar{P}}_{k}^{2}+{\hat{\tilde{x}}}_{k}^{2}{\left({\hat{\tilde{x}}}_{k}^{2}\right)}^{T}\right){\tilde{C}}_{22}^{T}$.

(48) $$\begin{array}{cc} \frac{\partial \left(tr\left({\bar{P}}_{f,k+1}\right)\right)}{\partial \left(K_{k}^{3}\right)} & =-2{\tilde{\rho }}_{1}\alpha_{1}\left(I-\alpha_{1}K_{k}^{3}F_{2}\right){\bar{P}}_{f,k}F_{2}^{T}+2{\tilde{\rho }}_{2}K_{k}^{3}{\tilde{C}}_{22}{\bar{P}}_{k}^{2}{\tilde{C}}_{22}^{T}+2{\tilde{\rho }}_{3}K_{k}^{3}S_{2}\beta^{2}S_{2}^{T}+2{\tilde{\rho }}_{4}\delta_{e}^{2}K_{k}^{3}\\ & +2\left(1+\rho_{11}\right)\alpha_{2}K_{k}^{3}{\tilde{M}}_{2}+2\left(1+\rho_{11}^{-1}\right)\alpha_{2}K_{k}^{3}{\tilde{M}}_{3}+2\left(1-\alpha \right)K_{k}^{3}{\tilde{V}}_{2}R_{v}{\left({\tilde{V}}_{2}\right)}^{T} \end{array}$$

where ${\tilde{\rho }}_{1}=1+\rho_{1}+\rho_{2}\alpha_{1}+\rho_{3}\alpha +\rho_{4}$, ${\tilde{\rho }}_{2}=\alpha_{1}^{2}+\rho_{2}^{-1}\alpha_{1}+\rho_{5}^{-1}\alpha_{1}+\rho_{8}\alpha \alpha_{1}+\rho_{9}\alpha_{1}$, ${\tilde{\rho }}_{3}=\alpha +\rho_{3}^{-1}\alpha +\rho_{6}^{-1}\alpha +\rho_{8}^{-1}\alpha \alpha_{1}+\rho_{10}\alpha$, ${\tilde{\rho }}_{4}=1+\rho_{4}^{-1}+\rho_{7}^{-1}+\rho_{9}^{-1}\alpha_{1}+\rho_{10}^{-1}\alpha$ and ${\tilde{M}}_{3}=F_{2}\left(\left(1+\rho_{12}\right){\bar{P}}_{f,k}+\left(1+\rho_{12}^{-1}\right){\hat{f}}_{k}{\hat{f}}_{k}^{T}\right)F_{2}^{T}$. Then, we have

(49) $$\begin{array}{cc} \alpha_{1}^{2}{\tilde{\tau }}_{1}K_{k}^{2}{\tilde{C}}_{22}{\bar{P}}_{k}^{2}{\tilde{C}}_{22}^{T} & +\alpha_{1}^{2}{\tilde{\tau }}_{2}K_{k}^{2}F_{2}{\bar{P}}_{f,k}F_{2}^{T}+{\tilde{\tau }}_{3}\delta_{e}^{2}K_{k}^{2}+{\tilde{\tau }}_{4}K_{k}^{2}S_{2}\beta^{2}S_{2}^{T}+\left(1-\alpha \right)K_{k}^{2}{\tilde{V}}_{2}R_{v}{\left({\tilde{V}}_{2}\right)}^{T}\\ & +\left(1+\tau_{14}\right)\alpha_{2}K_{k}^{2}{\tilde{M}}_{1}+\left(1+\tau_{14}^{-1}\right)\alpha_{2}K_{k}^{2}{\tilde{M}}_{2}=\alpha_{1}{\tilde{\tau }}_{1}{\tilde{A}}_{22}{\bar{P}}_{k}^{2}{\tilde{C}}_{22}^{T}+\alpha_{1}{\tilde{\tau }}_{2}{\hat{F}}_{2}{\bar{P}}_{f,k}F_{2}^{T} \end{array}$$

(50) $$\begin{array}{cc} {\tilde{\rho }}_{1}\alpha_{1}^{2}K_{k}^{3}F_{2}{\bar{P}}_{f,k}F_{2}^{T} & +{\tilde{\rho }}_{2}K_{k}^{3}{\tilde{C}}_{22}{\bar{P}}_{k}^{2}{\tilde{C}}_{22}^{T}+{\tilde{\rho }}_{3}K_{k}^{3}S_{2}\beta^{2}S_{2}^{T}+{\tilde{\rho }}_{4}\delta_{e}^{2}K_{k}^{3}+\left(1+\rho_{11}\right)\alpha_{2}K_{k}^{3}{\tilde{M}}_{2}\\ & +\left(1+\rho_{11}^{-1}\right)\alpha_{2}K_{k}^{3}{\tilde{M}}_{3}+\left(1-\alpha \right)K_{k}^{3}{\tilde{V}}_{2}R_{v}{\left({\tilde{V}}_{2}\right)}^{T}={\tilde{\rho }}_{1}\alpha_{1}{\bar{P}}_{f,k}F_{2}^{T} \end{array}$$

which can be further calculated as follows

(51) $$\begin{array}{cc} K_{k}^{2} & =\left(\alpha_{1}{\tilde{\tau }}_{1}{\tilde{A}}_{22}{\bar{P}}_{k}^{2}{\tilde{C}}_{22}^{T}+\alpha_{1}{\tilde{\tau }}_{2}{\hat{F}}_{2}{\bar{P}}_{f,k}F_{2}^{T}\right)\left(\alpha_{1}^{2}{\tilde{\tau }}_{1}{\tilde{C}}_{22}{\bar{P}}_{k}^{2}{\tilde{C}}_{22}^{T}+\alpha_{1}^{2}{\tilde{\tau }}_{2}F_{2}{\bar{P}}_{f,k}F_{2}^{T}+{\tilde{\tau }}_{3}\delta_{e}^{2}I+{\tilde{\tau }}_{4}S_{2}\beta^{2}S_{2}^{T}\right.\\ & {\left.+\left(1-\alpha \right){\tilde{V}}_{2}R_{v}{\left({\tilde{V}}_{2}\right)}^{T}+\left(1+\tau_{14}\right)\alpha_{2}{\tilde{M}}_{1}+\left(1+\tau_{14}^{-1}\right)\alpha_{2}{\tilde{M}}_{2}\right)}^{-1} \end{array}$$

(52) $$\begin{array}{cc} K_{k}^{3} & ={\tilde{\rho }}_{1}\alpha_{1}{\bar{P}}_{f,k}F_{2}^{T}\left({\tilde{\rho }}_{1}\alpha_{1}^{2}F_{2}{\bar{P}}_{f,k}F_{2}^{T}+{\tilde{\rho }}_{2}{\tilde{C}}_{22}{\bar{P}}_{k}^{2}{\tilde{C}}_{22}^{T}+{\tilde{\rho }}_{3}S_{2}\beta^{2}S_{2}^{T}+{\tilde{\rho }}_{4}\delta_{e}^{2}I\right.\\ & +\left(1+\rho_{11}\right)\alpha_{2}{\tilde{M}}_{2}{\left.+\left(1+\rho_{11}^{-1}\right)\alpha_{2}{\tilde{M}}_{3}+\left(1-\alpha \right){\tilde{V}}_{2}R_{v}{\left({\tilde{V}}_{2}\right)}^{T}\right)}^{-1} \end{array}$$

Hence, the desired filter gain matrices can be obtained via (51) and (52). In addition, the upper bounds ${\bar{P}}_{k+1}^{2}$ and ${\bar{P}}_{f,k+1}$ of the state and fault estimation error-covariance matrices are recursively calculated by Riccati-like difference Equations (35) and (36), respectively. ☐

Based on the results derived, the complete algorithm of the event-triggered remote fault estimation is concluded in Algorithm 2.

**Algorithm 2** Recursive algorithm of the event-triggered remote fault estimation.Set the initial conditions ${\bar{P}}_{0}^{1}$, ${\bar{P}}_{0}^{2}$, ${\bar{P}}_{f,0}$, ${\hat{\tilde{x}}}_{0}^{1}$, ${\hat{\tilde{x}}}_{0}^{2}$, $\gamma_{0}=1$ and k=0;
  1:**while** k≤N−1 **do**  2:    Calculate ${\bar{P}}_{k}^{1}$, ${\bar{P}}_{k}^{2}$ and ${\bar{P}}_{f,k}$ sequentially according to (19), (35) and (36);  3:    Calculate $K_{k}^{1}$, $K_{k}^{2}$ and $K_{k}^{3}$ in terms of (24), (45) and (46);    4:    **if** $∥{\tilde{y}}_{k}^{2}-{\tilde{y}}_{p,k}^{2}∥\leq \delta_{e}$ **then**  5:        $\gamma_{k}=0$, the remote fault estimator cannot receive the current measurement information to achieve energy conversation;  6:        State estimation step:  7:        ${\hat{\tilde{x}}}_{k+1}^{1}={\tilde{A}}_{11}{\hat{\tilde{x}}}_{k}^{1}+{\tilde{A}}_{12}{\hat{\tilde{x}}}_{k}^{2}+K_{k}^{1}\left({\tilde{y}}_{p,k}^{1}-{\hat{\tilde{y}}}_{k}^{1}\right)$;  8:        ${\hat{\tilde{y}}}_{k}^{1}=\alpha_{1}{\tilde{C}}_{11}{\hat{\tilde{x}}}_{k}^{1}$;  9:        ${\hat{\tilde{x}}}_{k+1}^{2}={\tilde{A}}_{22}{\hat{\tilde{x}}}_{k}^{2}+{\tilde{A}}_{21}{\hat{\tilde{x}}}_{k}^{1}+{\hat{F}}_{2}{\hat{f}}_{k}+K_{k}^{2}\left({\tilde{y}}_{p,k}^{2}-{\hat{\tilde{y}}}_{k}^{2}\right)$;10:        Fault estimation step:11:        ${\hat{f}}_{k+1}={\hat{f}}_{k}+K_{k}^{3}\left({\tilde{y}}_{p,k}^{2}-{\hat{\tilde{y}}}_{k}^{2}\right)$;12:        ${\hat{\tilde{y}}}_{k}^{2}=\alpha_{1}\left({\tilde{C}}_{22}{\hat{\tilde{x}}}_{k}^{2}+F_{2}{\hat{f}}_{k}\right)$;13:    **else**14:        $\gamma_{k}=1$, the current measurement information can be allowed to send out to ensure robust estimation;15:        State estimation step:16:        ${\hat{\tilde{x}}}_{k+1}^{1}={\tilde{A}}_{11}{\hat{\tilde{x}}}_{k}^{1}+{\tilde{A}}_{12}{\hat{\tilde{x}}}_{k}^{2}+K_{k}^{1}\left({\tilde{y}}_{k}^{1}-{\hat{\tilde{y}}}_{k}^{1}\right)$;17:        ${\hat{\tilde{x}}}_{k+1}^{2}={\tilde{A}}_{22}{\hat{\tilde{x}}}_{k}^{2}+{\tilde{A}}_{21}{\hat{\tilde{x}}}_{k}^{1}+{\hat{F}}_{2}{\hat{f}}_{k}+K_{k}^{2}\left({\tilde{y}}_{k}^{2}-{\hat{\tilde{y}}}_{k}^{2}\right)$;18:        Fault estimation step:19:        ${\hat{f}}_{k+1}={\hat{f}}_{k}+K_{k}^{3}\left({\tilde{y}}_{k}^{2}-{\hat{\tilde{y}}}_{k}^{2}\right)$;20:        ${\hat{\tilde{y}}}_{k}^{2}=\alpha_{1}\left({\tilde{C}}_{22}{\hat{\tilde{x}}}_{k}^{2}+F_{2}{\hat{f}}_{k}\right)$;21:    **end if**22:**end while**

**Remark** **7.** The scalars β, $\delta_{e}$ and $b_{d}$, reflected in the state and fault estimation error covariance upper bounds (35) and (36), represent the available items of randomly occurring deception attacks, event condition and fault difference, respectively. it is easily shown that the state and fault estimation error-covariance bounds are dependent on these scalars which means that bigger scalars β, $\delta_{e}$ and $b_{d}$ could lead to bigger upper bounds. It can be noted that the derivations of event-triggered state estimator for the 1st subsystem is not included in the proof of Theorems 3 and 4. They are omitted because their derivations are similar to the derivations of fault estimator for the 2nd subsystem, which have little influence on our main results. Furthermore, the performance of the proposed fault estimator coincides with that of the time-driven estimator for $\delta_{e}=0$. Hence, the derivation of event-triggered fault estimator is omitted, when $\gamma_{k}=1$.

**Remark** **8.** From the event condition in (37), it is clear that the proposed event-triggered sensor data-transmission scheme is based on a send-on-delta regulation [30,31], namely, only when the measurement values change more than a predetermined threshold, the sensors transmit their sampling data to remote data centers for processing. Neither the approximated probability density functions of states conditional on measurements nor the assumption on the distribution of $\delta_{k}$ is required in the presented fault estimator. In addition, the implementation of event-triggered scheme cannot bring too much computational burden, which is proved in the next section. The design of fault-detection filter and fault estimator are simple because they are based on reduced-order subsystems. In other words, the applicability and feasibility of the event-triggered fault estimator are enhanced.

## 5. Experimental Verification

### 5.1. Experimental Setup

**Figure 2:**
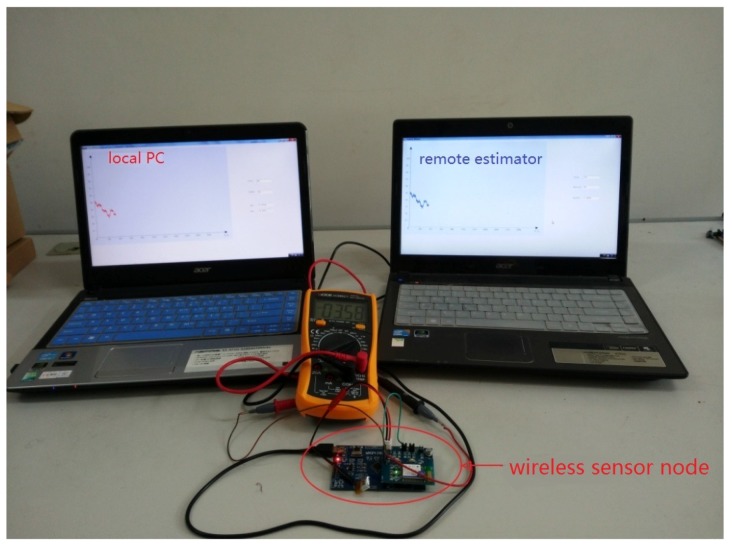
The components of our system.

To evaluate the performance of the designed fault-detection strategy and event-triggered fault estimator, a simulation platform, representing a scaled version of an industrial system, is implemented, where the terminal voltages of a three-cell battery string are observed over a wireless channel. As shown in Figure 2, the dynamic system which worked in a local personal computer which collocated with a sensor node communicates wirelessly with a remote estimator. We now describe the details of the components of our system.

**Figure 3:**
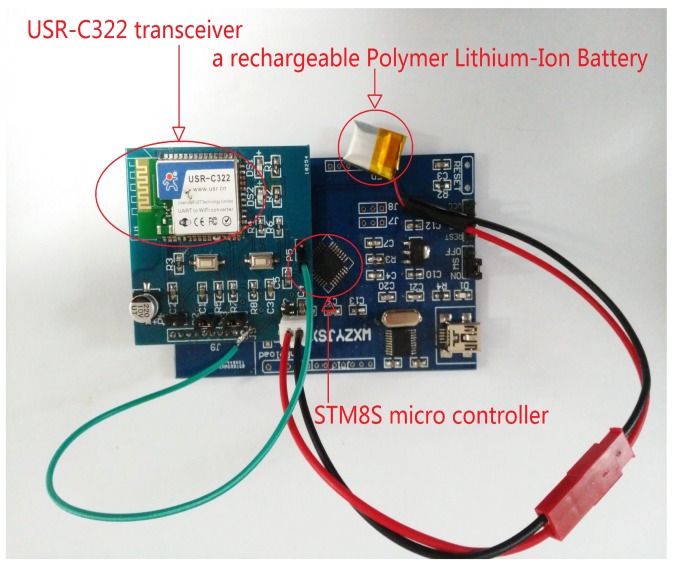
The components of our wireless sensor node.

Figure 3 shows that hardwares of wireless sensor node that consist of an STM8S micro-controller, USR-C322 transceiver and a rechargeable polymer lithium-ion battery. The reason why we choose the wireless transceiver is that it can use a low power consumption pattern. Indeed, most wireless modules belong to the characteristic of high performance in the mainstream market. Despite the fact that event-triggered transmission mechanism can make the wireless modules sleep, it still consumes the energy similarly as it works. Luckily, this problem has been successfully solved through choosing USR-C322. Then, it makes the module enter a “status of deep sleep” and stop working completely. Moreover, STM8S microcontroller has embedded 32 Kbyte Flash, 2 Kbyte RAM, 16-bit advanced control timer and abundant communication interfaces that allow us to use them as a sensor node. The selection of STM8S microcontroller implies that the implementation of event-triggered scheme cannot bring too much computational burden. More information about the hardwares of the node can be found in [32,33].

Now, the proposed theoretical results is applied to a linear continuous-time system of the three-cell battery string presented in [11]. After discretisation with sampling period T=1s, the discrete-time system can be described as the system (1) with following parameters

(53) $$A=\left[\begin{array}{ccc} -0.6026 & 0 & 0\\ 0 & -0.6026 & 0\\ 0 & 0 & -0.6026 \end{array}\right],\;C=\left[\begin{array}{ccc} 1 & 0 & 0\\ 0 & 1 & 0\\ 0 & 0 & 1 \end{array}\right],\;B=\left[\begin{array}{ccc} 0.1795 & 5.2484 & 0.0225\\ 0.1795 & 5.2484 & 0.0225\\ 0.1795 & 5.2484 & 0.0225 \end{array}\right]$$

(54) $$W=\left[\begin{array}{c} -3\\ 1\\ -1 \end{array}\right],\;\;V=\left[\begin{array}{c} 0\\ 1\\ 1 \end{array}\right],\;\;D=\left[\begin{array}{c} 2\\ 1\\ 1 \end{array}\right],\;\;\bar{F}=\tilde{F}=\left(\begin{array}{c} 3\\ 2\\ 1 \end{array}\right)$$

where $x_{k}=\left[\begin{array}{ccc} V_{k}^{1} & V_{k}^{2} & V_{k}^{3} \end{array}\right]$ and $V_{k}^{i}$ are the terminal voltage of each cell of the ith cell. Control input of this batter system $u_{k}=\left[\begin{array}{ccc} Z & 1 & C_{u} \end{array}\right]$ in which Z is state of charge and the current $C_{u}=3A$. Considering the main technical specifications of voltage sensors, the following parameters are chosen as $Q_{w}=0.2915$ and $R_{v}=0.3606$. For k=0,1,…N−1 and N=100, the unknown disturbances $d_{k}$ are supposed to be a random noise uniformly distributed in $\left[-0.32,0.32\right]$.

In order to transform the original system, the following transform matrices *S* and *T* are used as

(55) $$S=T=\left[\begin{array}{ccc} 1 & 0 & -2\\ 0 & 1 & -2\\ 0 & 0 & 1 \end{array}\right]$$

Then, we have

(56) $$TAT^{-1}=\left[\begin{array}{ccc} -0.6026 & 0 & 0\\ 0 & -0.6026 & 0\\ 0 & 0 & -0.6026 \end{array}\right],\;TB=\left[\begin{array}{ccc} -0.1795 & -5.2484 & -0.0225\\ -0.1795 & -5.2484 & -0.0225\\ 0.1795 & 5.2484 & 0.0225 \end{array}\right]$$

(57) $$TW=\left[\begin{array}{c} -1\\ 3\\ -1 \end{array}\right],\;TD=\left[\begin{array}{c} 0\\ -1\\ 1 \end{array}\right],\;T\tilde{F}=S\bar{F}=\left[\begin{array}{c} 1\\ 0\\ 1 \end{array}\right],\;SCT^{-1}=\left[\begin{array}{ccc} 1 & 0 & 0\\ 0 & 1 & 0\\ 0 & 0 & 1 \end{array}\right],\;SV=\left[\begin{array}{c} -2\\ -1\\ 1 \end{array}\right]$$

The probability of deception attacks, the upper bound of fault difference item and the event-triggered transmission threshold are selected respectively as: α=2%, $b_{d}=0.45$ and $\delta_{e}=0.017$. Furthermore, $\tau_{j}\left(j=1,\;\ldots ,\;15\right)$ are chosen as 1, *β* and $\rho_{j}\left(j=1,\;\ldots ,\;12\right)$ are determined as 0.15.

### 5.2. Experimental Results

The experiment consisting of four parts are designed to verify the effectiveness of the obtained theoretical results: (A) the performance on the remote state estimation without faults and fault-detection strategy; (B) the accuracy on the remote fault estimation and comparsion between a learning observer [11,34] and our proposed algorithm; (C) the analysis on the energy-saving trend of a 50 mAh polymer lithium-ion battery and (D) the effect on remote event-triggered fault estimator with the increased probabilities of deception attacks.

**Figure 4:**
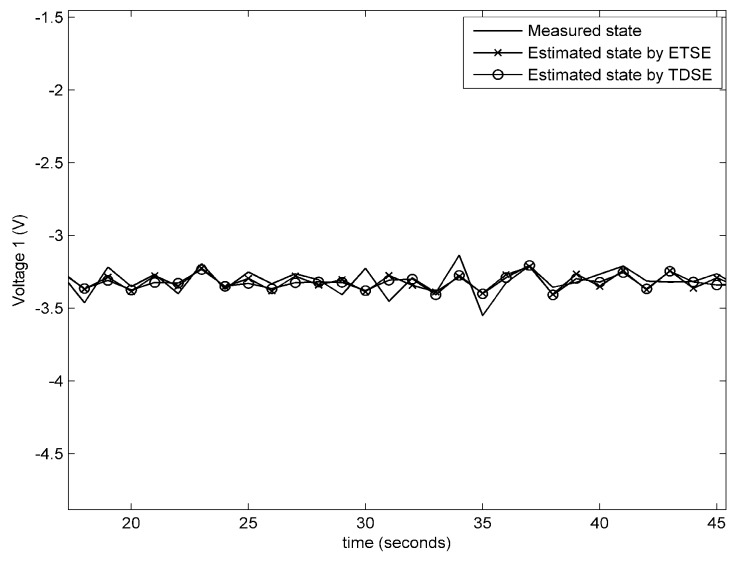
Estimated and measured voltages for the 1st battery cell.

**Figure 5:**
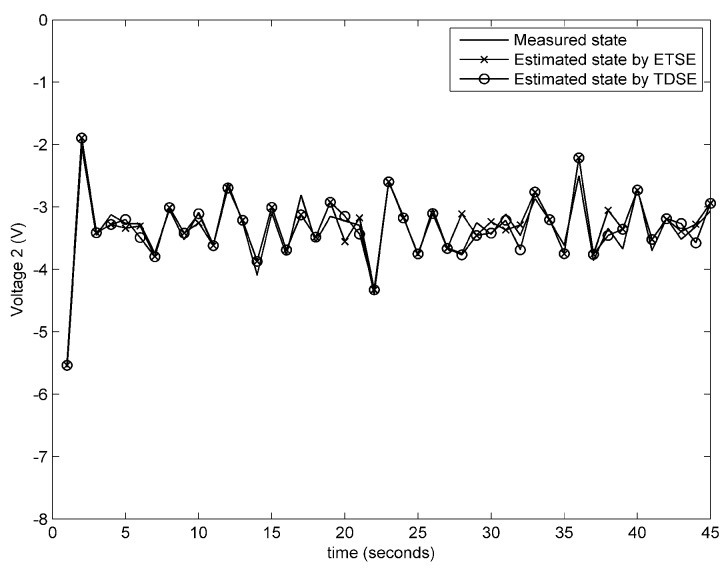
Estimated and measured voltages for the 2nd battery cell.

**Figure 6:**
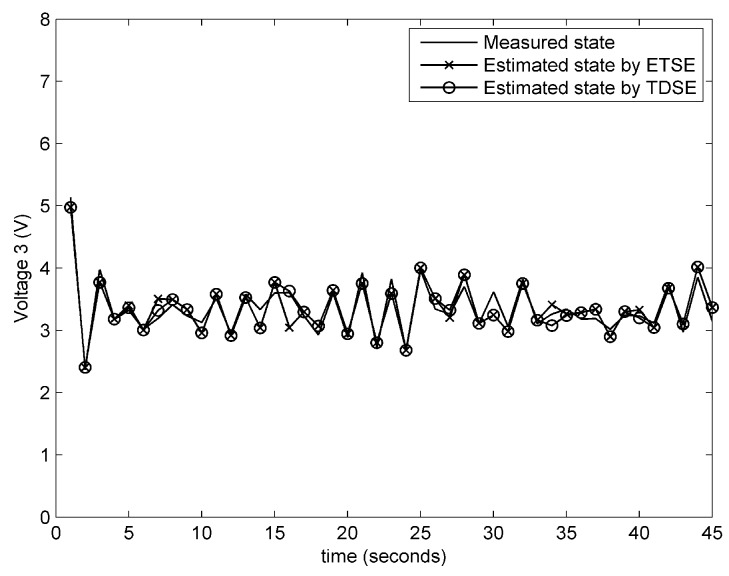
Estimated and measured voltages for the 3rd battery cell.

**Figure 7:**
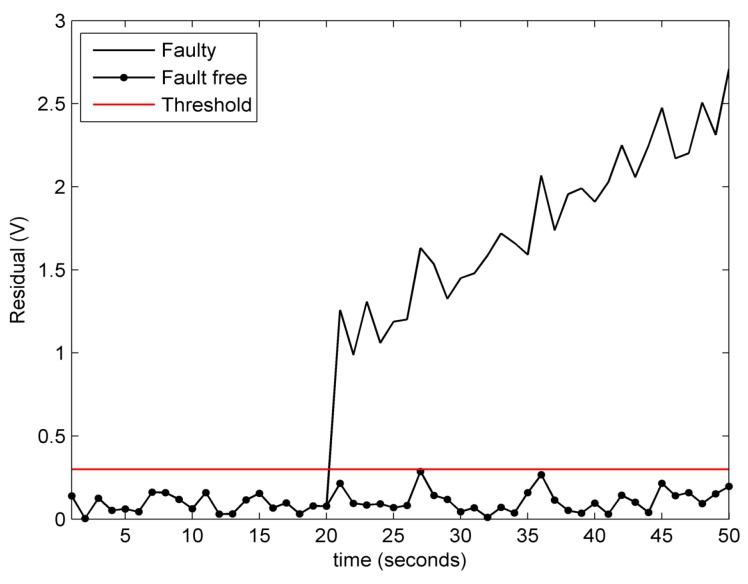
Fault-detection residual of the 1st subsystem.

**Experiment** **1.** *In the first experiment, the information sent by attacker is $\beta_{k}={\left[\begin{array}{ccc} 0.1e^{-0.5k} & 0.13\sin k & 0 \end{array}\right]}^{T}$, which is also applied to the subsequent experiments. The performance on remote state estimation with fault-free condition and fault-alarming strategy are verified in Figure 4, Figure 5, Figure 6 and Figure 7. As shown in Figure 4, Figure 5 and Figure 6, the estimated voltages by an event-triggered state estimator (ETSE) approximately closes to the measured trajectories and the estimated voltages by a time-driven state estimator (TDSE), leading to a accurate state estimation result. Next, assume that a unknown fault is created as follows:*

(58) $$f_{k}=\left\{\begin{array}{cc} 0 & k<19\\ 0.1k & otherwise \end{array}\right.$$

In Figure 7, the red line at 0.5 V is the threshold $\delta_{f}$ that we selected for fault alarming. When a fault occurs at 20 s, fault-detection residual $r_{k}$ is quickly diverging. Comparatively, the residual $r_{k}$ with no fault still remains a convergence status. It is clear that fault can be detected immediately via the proposed fault-alarming strategy.

**Experiment** **2.** *Here, the effectiveness of event-triggered fault estimator (11) is evaluated in the presence of randomly occurring deception attacks. Let us consider a constant fault and a time-varying fault are respectively supposed as*

(59) $$f_{k}=\left\{\begin{array}{c} 5\;\;\;\;\;k<50\\ -5\;\;otherwise \end{array}\right.\;\mathrm{and}\;f_{k}=\left\{\begin{array}{c} 0.7\sin\left(0.5k\right)\;\;\;\;\;\;\;\;k<50\\ -\sin\left(0.5k\right)-0.5\;\;otherwise \end{array}\right.$$

**Figure 8:**
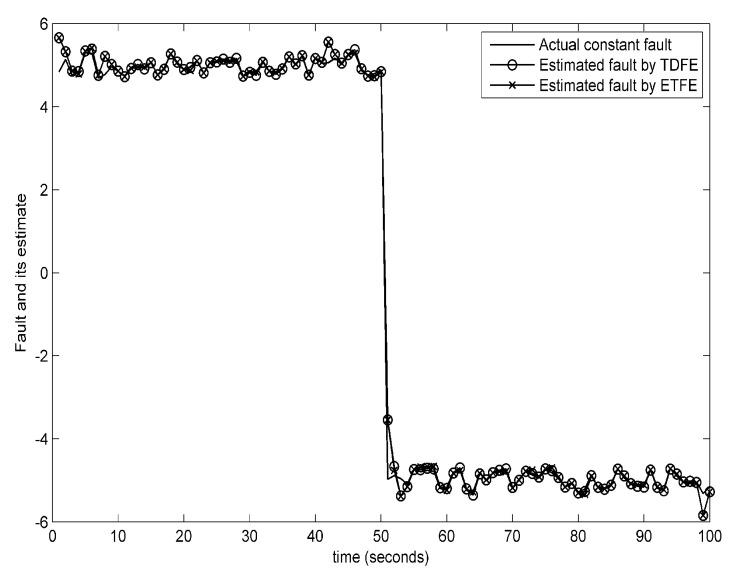
Fault estimation of the constant fault.

**Figure 9:**
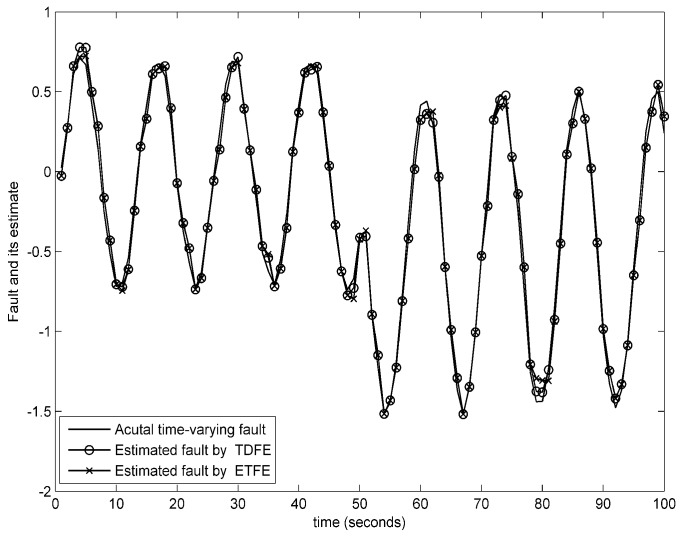
Fault estimation of the time-varying fault.

**Figure 10:**
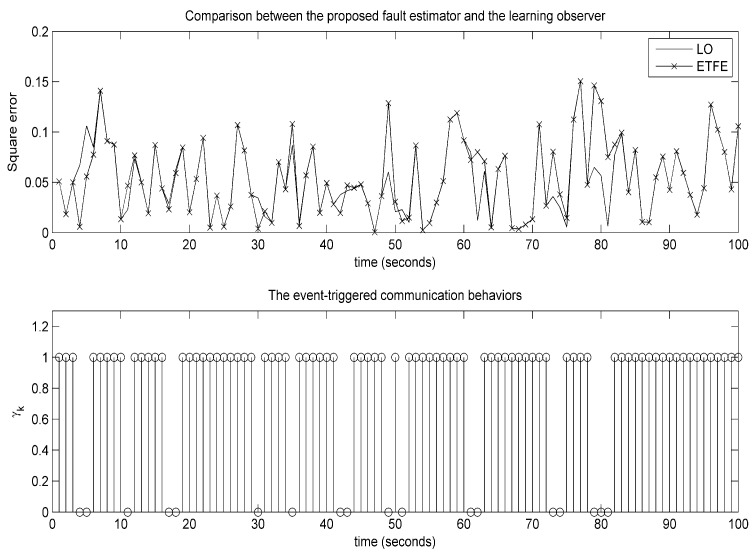
The evolution of square error and the corresponding event-triggered communication behaviors.

The estimation trajectories of constant and time-varying faults using the presented event-triggered fault estimator (ETFE) and time-driven fault estimator (TDFE) are depicted in Figure 8 and Figure 9. It is observed that the proposed estimation algorithm has the ability to robustly construct constant and time-varying faults. Further, to compare the fault estimation performance clearly, the square error ${\left(f_{d,k}-{\hat{f}}_{k}\right)}^{T}\times \left(f_{d,k}-{\hat{f}}_{k}\right)$ on actual time-varying fault described in (59), the estimated fault computed by a periodic learning observer (LO) and our fault estimation algorithm over 1000 Monte Carlo runs are demonstrated in Figure 10. The corresponding communication behaviors of event-triggered sensor transmission scheme are also presented in Figure 10. It is indicated that the square error using the proposed estimation algorithm is slightly larger than the learning observer. From these figures, it can be concluded that the estimation performance is not decreased obviously although the obtained event-triggered strategy reduces the transmission times relatively.

**Figure 11:**
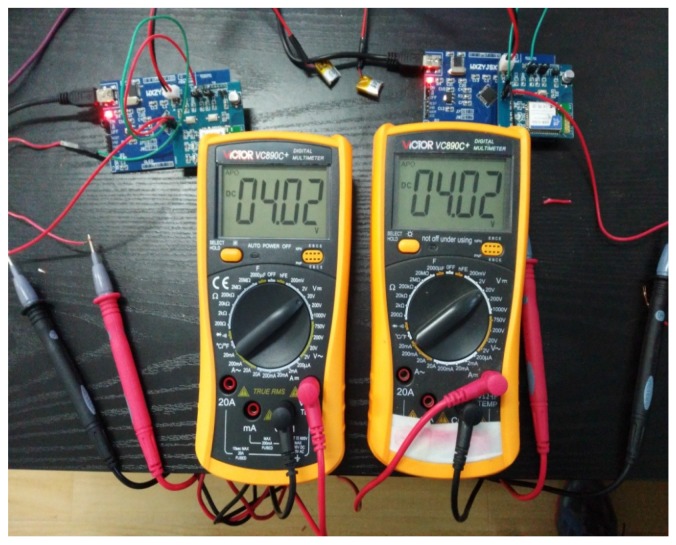
The initial voltages of Polymer Lithium-Ion batteries.

**Figure 12:**
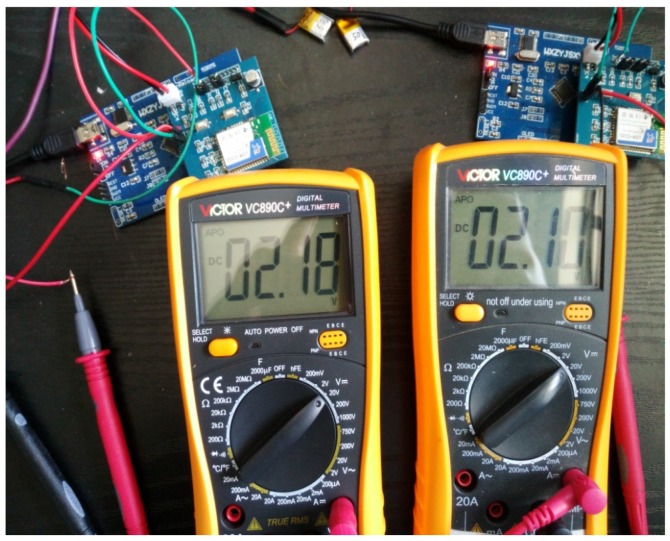
The final voltages of Polymer Lithium-Ion batteries.

**Figure 13:**
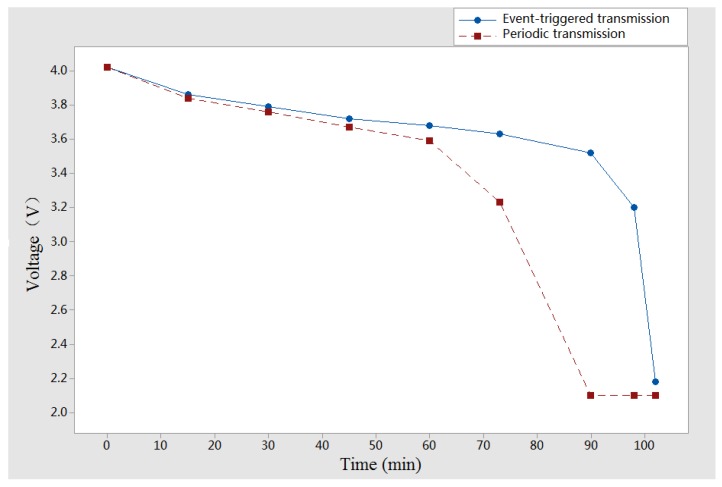
The relationship between time and voltages.

**Experiment** **3.** In order to investigate the proposed event-triggered transmission scheme which influences the battery life, we use a 50 mAh Polymer Lithium-Ion battery to complete our third experiment. As shown in Figure 11, the batteries have been fully charged when we get ready to run the procedure, while the wireless sensor nodes have not worked in Figure 12 since the batteries were run out. The relationship between time and 50 mAh battery voltages is given in Figure 13, which is shown to compare with periodic data transmission mechanism, the proposed event-triggered sensor data transmission scheme can prolong about 11.8% battery life.

**Figure 14:**
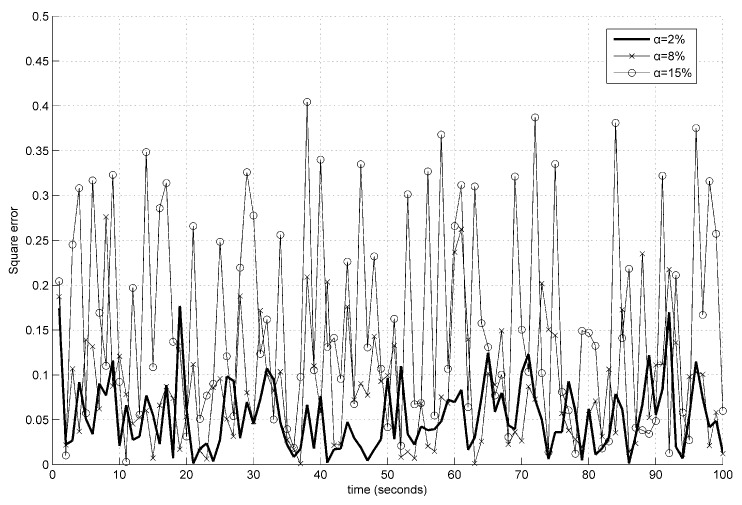
The evolution of square estimation error with the increased probabilities of deception attacks.

**Experiment** **4.** In the final experiment, the effect on remote fault estimator accuracy is examined in the presence of the increased probabilities of deception attacks. Similar to the second experiment, the square estimation error ${\left(f_{d,k}-{\hat{f}}_{k}\right)}^{T}\times \left(f_{d,k}-{\hat{f}}_{k}\right)$ on actual time-varying fault borrowed from (59), the estimated fault calculated by the proposed fault estimation algorithm are given in Figure 14. It can be found that a bigger attack probability results in a bigger error bound, which implies that estimation performance degrades slightly as the attack probability increases.

## 6. Conclusions and Future Work

This paper presented a synthesized design of two types of estimators to simultaneously event-triggered fault detection and fault estimation for a class of discrete-time stochastic systems subject to subject to unknown disturbances and randomly occurring deception attacks. The unknown disturbances were removed from the 1st subsystem using a coordinate transformation approach so as to ensure the sensitivity of fault detection. An upper bound of fault-detection filtering error covariance matrix was recursively calculated by a Riccati-type difference equation. The explicit form of the fault-detection filtering gain was obtained to minimize such upper bound through a recursive algorithm. In order to achieve real-time capacity of fault-detection, a novel fault-alarming framework was considered which could effectively solve a time-delay issue of fault alarming when an event condition was triggered. Similar to the design of fault-detection filter, fault estimator was designed so as to derive the upper bounds of state and fault estimation error covariance matrices and minimize them at each time step. At the end of paper, a simulation platform, where the terminal voltages of a three-cell battery string was estimated over a wireless channel, was verified to the feasibility and effectiveness of the proposed theoretical results. In the future, the proposed results will be extended to some more practical cases including more general time-varying nonlinear systems and multi-sensor scheduling problems.
